# Approximating Cognitive Representations Using Space

**DOI:** 10.1162/OPMI.a.389

**Published:** 2026-10-03

**Authors:** John R. Starr, Ashlyn Winship, Marten van Schijndel

**Affiliations:** Department of Cognitive Science, Johns Hopkins University, Baltimore, MD, USA; Department of Linguistics, Cornell University, Ithaca, NY, USA

**Keywords:** representations, psycholinguistics, acceptability, typicality, similarity, paradigm

## Abstract

In everyday life, people intuitively use space to make meaningful distinctions between objects. In this paper, we demonstrate how geometric, spatial representations can capture a wide variety of cognitive phenomena. To conduct these experiments, we developed GRIS (Generating Representations In Space), an advancement of prior spatial-arrangement paradigms. In GRIS experiments, participants manipulate a set of objects (text, audio, images) and place them on canvases. Following an introduction to the paradigm, we present three studies which demonstrate how experiments in the GRIS paradigm can both a) reveal nuanced insights about human and computational representations, and b) capture low-level behavioral data of spatial representations as they are constructed.

## INTRODUCTION

In our daily lives, we use space to make and represent meaningful relationships between objects: we separate different kinds of clothes into different compartments, read menus that spatially group items on the page according to their broader classifications, and press elevator buttons that are vertically ordered to reflect the structure of their buildings. In these ways and many more, humans intuitively use space to simplify choice, perception, and computation (Kirsh, 1995), allowing us to navigate and represent complex structures and relations with ease.

One of the primary approaches to studying space in the cognitive sciences is through its relationship with language. Previous research has shown that people construct mental representations of words that encode spatial relationships (Bryant, 1997; Kemmerer, 1999; Taylor & Tversky, 1992), and that these relationships are marked on a schematic level of varying detail (Hayward & Tarr, 1995; Landau & Jackendoff, 1993; Talmy, 1983; Tversky & Lee, 1998). Studies in this domain often focus on how language organizes our cognitive representations of objects and their locations, both in discourse and in the real world. Other work on the relationship between space and language suggests that we transfer linguistic information onto mental spaces of the world (consisting of information about referents, their beliefs, actions, etc.), which we then blend together to understand the relevant discourse (Fauconnier, 1994, 2010; Sweetser, 1999).

In this paper, we advance an alternative perspective on the relationship between space and language: space as a tool to contextualize our understanding of language, and, more broadly, human cognition (Goldstone, 1994; Hout et al., 2013; Kriegeskorte & Mur, 2012). To show the utility of this alternative perspective, we present an experimental paradigm—GRIS (**G**enerating **R**epresentations **I**n **S**pace)—which (a) capitalizes on the way humans intuitively use space, (b) approximates representations of language and other cognitive phenomena, and (c) does so in a way that is easily comparable to embedding representations from computational models, allowing us to further probe the matches and mismatches between humans and models. Similar to other spatial arrangement methods, participants in GRIS experiments move objects (text, image, audio) onto a canvas and use space in a meaningful way; however, unlike prior methods, GRIS collects incremental information about which objects were moved, when they were moved, and where they moved to. To briefly highlight our results, we demonstrate how space can be meaningfully used to (1) both replicate findings from other psycholinguistic paradigms and provide further contextual nuance to such results, (2) develop multi-dimensional graphs that can be used for computational modeling, and (3) provide insights into incremental cognitive decisions that inform our understanding of human behavior. More broadly, we argue that GRIS allows participants to use their natural intuitions about space to inform our understanding of how people represent various kinds of linguistic information.

### Article Organization

In the following section, we provide further motivation for developing a paradigm that uses space meaningfully. In GRIS: A Walkthrough section, we outline the GRIS paradigm, introducing its key functionalities and structure, along with a brief case study showing how it can be used. In Experiment 1: Multi-Dimensional Similarity to Experiment 3: Sentence Acceptability sections, we present three GRIS experiments,^1^ demonstrating how the paradigm can (a) replicate prior results across a number of cognitive domains, and (b) capture more nuanced relationships between representations than other experimental paradigms and computational models of linguistic structure.^2^ In General Discussion section, we discuss the general implications of GRIS and present possible directions for future work.

## ON SPACE

### Space and Psycholinguistic Paradigms

From a design perspective, many standard psycholinguistic paradigms^3^ minimally engage with space: standard rating and judgment tasks often present an item in isolation (or near isolation) alongside a scale or a drop-down box, and forced-choice tasks only capture the pairwise differences between one or two items. Some experimental paradigms do inherently use space as a metric for psycholinguistic effort, such as measuring how participants’ eyes move to different locations on a screen when using eye-tracking in the visual world paradigm (Cooper, 1974; Tanenhaus et al., 1995), or following the trajectory of a participant’s mouse/cursor across the screen using a mouse-tracking paradigm (Freeman & Ambady, 2010; Wilcox et al., 2024). These paradigms usefully capture various cognitive processes: for example, researchers have used spatial data from mouse-tracking data to probe how people make decisions (Maldonado et al., 2019) and process complex syntactic structures (Farmer et al., 2007).

Other experimental paradigms use space in a way that geometrically captures internal representation spaces. For example, Goldstone (1994) proposed the Spatial Arrangement Method (SpAM), an experimental paradigm where participants freely arrange items on a plain 2D screen according to their similarity; at the end of each trial, more similar objects should be placed closer together to form a complete weighted graph. Goldstone (1994) found that SpAM effectively captures similarity judgments for visual stimuli. Later work involving SpAM has shown that SpAM results strongly correlate with pairwise judgments for both adults (Hout et al., 2013) and children (Unger et al., 2016), and that spatial methods can capture high-dimensional semantic spaces for a variety of stimuli and research questions (Richie et al., 2020). Kriegeskorte and Mur (2012) demonstrated that extending the single-arrangement approach of SpAM to a multi-arrangement method—where participants first arrange all the objects in space, and then re-arrange particular subsets of the data—allows researchers to capture high-dimensional similarity spaces from the final positions of multiple 2D arrangements.

Additionally, spatial arrangement methods have been shown to be more efficient than other experimental methods, such as pairwise judgments (Hout & Goldinger, 2016; Hout et al., 2013). As an example, consider a rating task where a participant is asked to rate the difference between item pairs on a scale (according to some metric), for a total of four unique items. We visualize two possible iterations of this experiment in Figure 1.

**Figure 1.**
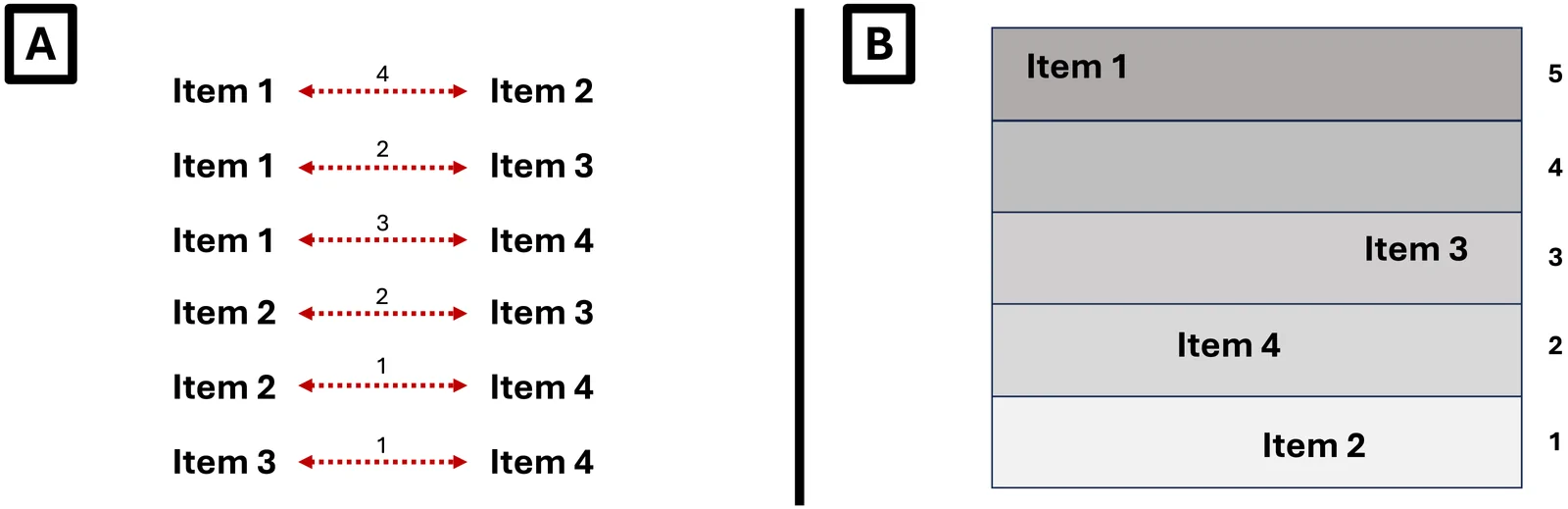
Sample rating tasks for four items. (A) Ratings in a pairwise comparison layout, where the number above the arrows reflects the scalar differences between two items. (B) Ratings in a space-motivated layout. The overall scalar ratings are identical between (A) and (B).

In Figure 1A, participants are asked to directly measure the difference between all possible combinations of the four items (either in one trial or across several trials). For these items, participants maintain six^4^ pairwise comparisons that are all in relation to one another. Alternatively, the experiment in Figure 1B presents a version of the experiment that capitalizes on human spatial intuitions: participants are asked to use space to distinguish between all possible combinations, where larger separation between items reflects larger differences. Note that the absolute value of the ratings are identical between both iterations of the experiment.

In alignment with prior work, we argue that there are two primary advantages to Experiment (B) over Experiment (A) (Goldstone, 1994; Hout et al., 2013; Kriegeskorte & Mur, 2012). First, participants are able to concretize the relative relations. Rather than needing to maintain an implicit (and potentially inconsistent) scale of differences as in Experiment (A), in Experiment (B) the relations between items are explicitly visualized in a manner that is easy to directly manipulate and update on a global level.^5^ Second, Experiment (B) contextualizes the different items and their relative relations, allowing participants to quickly set the bounds of the underlying scale(s) that they are using to distinguish between items.

### Space and Computational Models

Space has also been used to approximate different cognitive representation spaces in computational models. For example, vector spaces that are generated by modern computational models of language are often used as interpretable proxies for human linguistic structure: for example, high-dimensional vectors for the words “cat” and “dog” are typically near one another in computational vector spaces, whether such vectors are computed using word co-occurrence statistics (e.g., Pennington et al., 2014) or more complex, contextual operations (e.g., Radford et al., 2019). Accordingly, close proximity in computational vector spaces^6^ is often interpreted as human-like similarity^7^ at all levels of linguistic structure, including phonetic information (Parrish, 2017; Zouhar et al., 2023), phonological segments (Silfverberg et al., 2018), phrases (Passos et al., 2014), and others.

However, while this interpretation about the relationship between human and model representations holds true generally, prior work has noted some mismatches: for example, model representations have been shown to occupy a narrow region of the embedding space (a phenomenon known as *anisotropy*; Ethayarajh, 2019; Mimno & Thompson, 2017), have “rogue” dimensions that dominate similarity metrics (Timkey & van Schijndel, 2021), and their representations fail to be robust to minor orthographic noise (Matthews et al., 2024).^8^ Moreover, to our knowledge, no prior work has used spatial representations to approximate human representational spaces of linguistic data in a manner that is comparable to those generated by off-the-shelf computational models.

## GRIS: A WALKTHROUGH

The core idea of the GRIS paradigm is to provide participants with objects that they can drag and drop onto a labeled canvas. In the following two subsections, we overview the structure of a GRIS experiment and demonstrate how participants navigate through a trial.

### Structuring a GRIS Experiment

GRIS experiments have two fundamental components: (1) objects that can be placed, and (2) a canvas to place objects on.

Objects can be text, images, or audio; these objects are distinct targets that can be individually moved.^9^ By default, objects are located in a reservoir at the bottom of the screen, though their initial positions can be changed to accommodate relevant research questions.

Canvases, from a participant’s perspective, can be either blank or split into different categories; some sample canvases are provided in Figure 2. From a researcher’s perspective, canvases are built of individual, labeled canvas blocks that are either square or rectangular. Unlike prior spatial arrangement paradigms which use 2D coordinates, canvas blocks are labeled using a four-point coordinate system (*x-cat*, *y-cat*, *x-abs*, *y-abs*), where the first two dimensions are used to mark the broader group that the block belongs to (as in the categorical canvases B & C in Figure 2), and the last two dimensions are used to mark the absolute position of the block on the overall canvas; note that canvas labels can be modified to accommodate other systems (i.e., word labels). Beyond labeling, each canvas block can be independently specified for height, width, and color. Finally, images can be overlaid on the canvas, allowing for additional designs beyond those possible by combinations of squares and rectangles.

**Figure 2.**
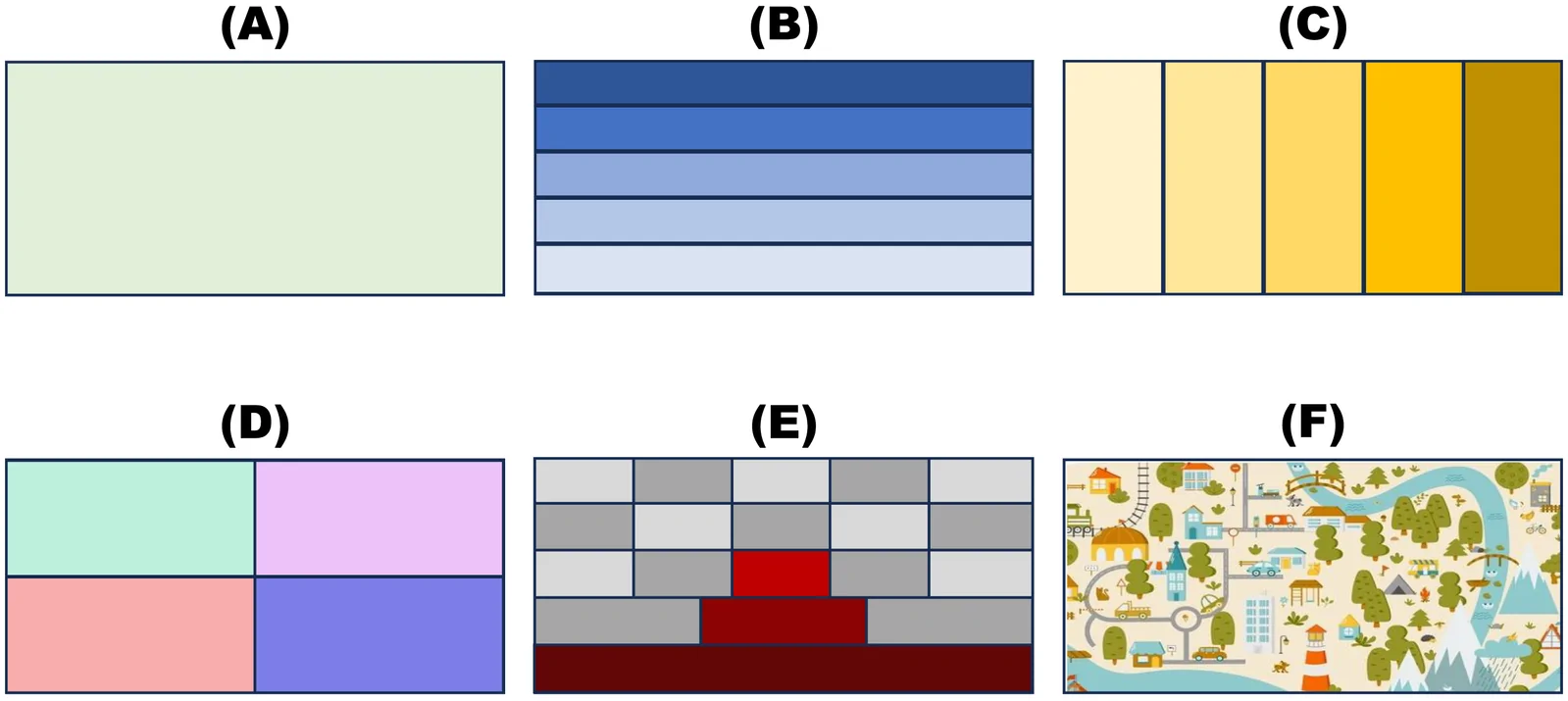
Sample GRIS canvases. Canvases can be blank (A), split into categories in both cartesian dimensions (B, C) simultaneously (D) and irregularly (E), or placed under an image (F).

For ease of use for other researchers, we have developed the GRIS toolkit,^10^ which provides instructions on how to build, run, and analyze GRIS experiments.

### Participating in a GRIS Experiment

GRIS experiments are designed to be simple and intuitive for participants. To explain how a participant navigates through a GRIS experiment, we provide a sample, partially-completed trial in Figure 3. In this sample trial, the participant has access to five objects—different shapes—which begin in the reservoir (B) below the blank canvas (A). The instructions at the top of the screen indicate that the participant should order the objects in a line, where the leftmost shapes are the “roundest” according to their intuitions. The participant first placed the star on the right boundary of the screen, then placed the oval on the left boundary; the abnormal shape was placed between these two shapes. Once they have placed all five objects on the canvas, the participant will be prompted to continue to the next trial, though they can continue to re-arrange the objects at any point in time throughout the trial.

**Figure 3.**
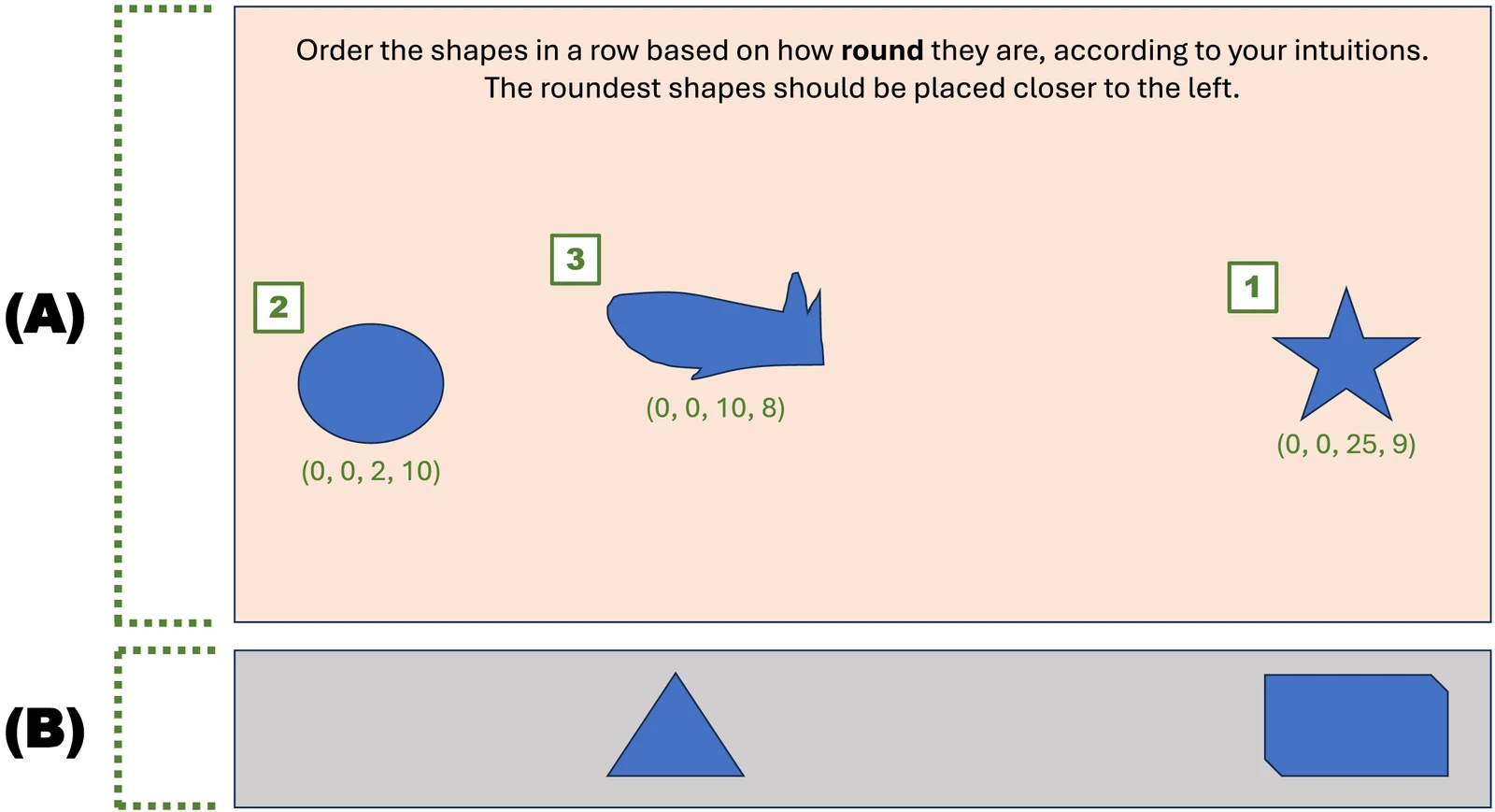
Sample of a partially-completed GRIS trial. Participants see a canvas (A) and a reservoir of objects (B); participants do not see anything marked in green. In this example, the objects are shapes. Participants are presented instructions—either during the trial (as in this figure) or prior to the trial—which guide how the participant should manipulate the objects. For each recorded drag-and-drop action, data is collected about the time/order of that action (boxed green numbers in the figure) and both the original and new coordinates for that object; only the dropped coordinates are presented in the figure.

For each drag-and-drop, GRIS collects (1) which object was moved, (2) the object’s original location, (3) its new location, and (4) the timestamps for both the initial drag and the final drop. Data are also collected about when each trial begins and ends, as well as the final positions for all objects at the conclusion of each trial.

### Case Study: Incremental Determination of Category Membership

To demonstrate one way to analyze participants’ incremental updates to item placements during trials, we present a brief case study drawn from (ANONYMIZED)^11^ on how people categorize polysemous words; for example, “bank” may refer to a feature of a river, or to a public lending institution (Basili et al., 1997; Oden & Spira, 1983; Rodd, 2020).

In this case study, we recruited 69 participants on Prolific and paid them at an approximate rate of $12 per hour. Participants were presented with a blank canvas, and told to organize words according to how similar they find them. Words appear in the word bank below the canvas in sequence, and participants must place the current word on the canvas before the next word becomes available. Participants are able to adjust the position of any word at any point during the trial.

#### Items.

An experimental trial consists of seven words: a polysemous word which may belong to two distinct categories (the Target), three words belonging to Category 1, and three words belonging to Category 2. We looked at two polysemous words and their associated categories; see Table 1.

**Table 1.** Subset of experimental items.

Target	Category 1	Category 2
bank	river feature:	institution:
	waterfall, rapids, floodplain	post office, library, hospital
plant	manufactory:	biota:
	factory, mill, forge	bacteria, fungus, animal

Words belonging to Category 1 are presented before those belonging to Category 2. Each category gets its own turn as Category 1 or 2, so that if river feature is Category 1 for one participant, it will be Category 2 for another participant. The Target is presented in one of three Positions. In Position 1, it is presented before any words belonging to Category 2 are shown. In Position 2, it is presented after at least one word from Category 2 has been revealed, but before the final word. In Position 3, the Target is presented as the final word. If participants are sensitive to the new context that is introduced by the Category 2 words, they should update their Position 1 Target placement once the Category 2 words start being presented. Finally, at the end of a trial, participants are presented with an end prompt that reads: “Ensure you have placed the objects where you want them, and then please hit the button below to continue.” This prompt confirms that the final spatial organization of items on the canvas reflects participants’ intended relationships between items.

We also conducted control trials, in which a Target is presented alongside words from *either* Category 1 or Category 2, and words from an unrelated category. For example, to test its association with the category river feature, “bank” was presented with the words from that category and words from the unrelated category tree: [oak, pine, maple].^12^ In the control trials, the category assignment of the Target should be unambiguous, so participants should place it closer to the Category 1/Category 2 items, and further from the items belonging to the unrelated category. The control trials allowed us to measure the baseline distance of the Target from each of its potential categories.

#### Results.

Participants update their spatial representation of the Target in response to ambiguous (experimental) trials. The distance of the Target from a category is determined by calculating the Euclidean distance of the Target from the center of the cluster formed by the other words in the category. In the unambiguous control trials, the Target is placed closer to the single related category that is available; in the ambiguous experimental trials, it is placed further from both available categories (Figure 4). This suggests that the spatial relationship between the Target and other words is not fixed, but is instead updated to reflect ambiguous contexts (i.e., “bank” is placed closer to “rapids” in an unambiguous context, but further from “rapids” in an ambiguous context where river feature is not the only possible category for “bank”). These results are further supported by the data shown in Figure 5. In trials where the Target is presented in Position 1, participants are willing to update the Target’s placement after being exposed to the words from Category 2, showing that when new context is presented, participants will re-categorize the Target to reflect ambiguous category membership. Additionally, throughout the trial, participants are updating the placement of the Target, and they also do so after being presented with the end prompt. This shows that the participants do not consider the spatial relationships between items to be static, but instead are continually updating them.

**Figure 4.**
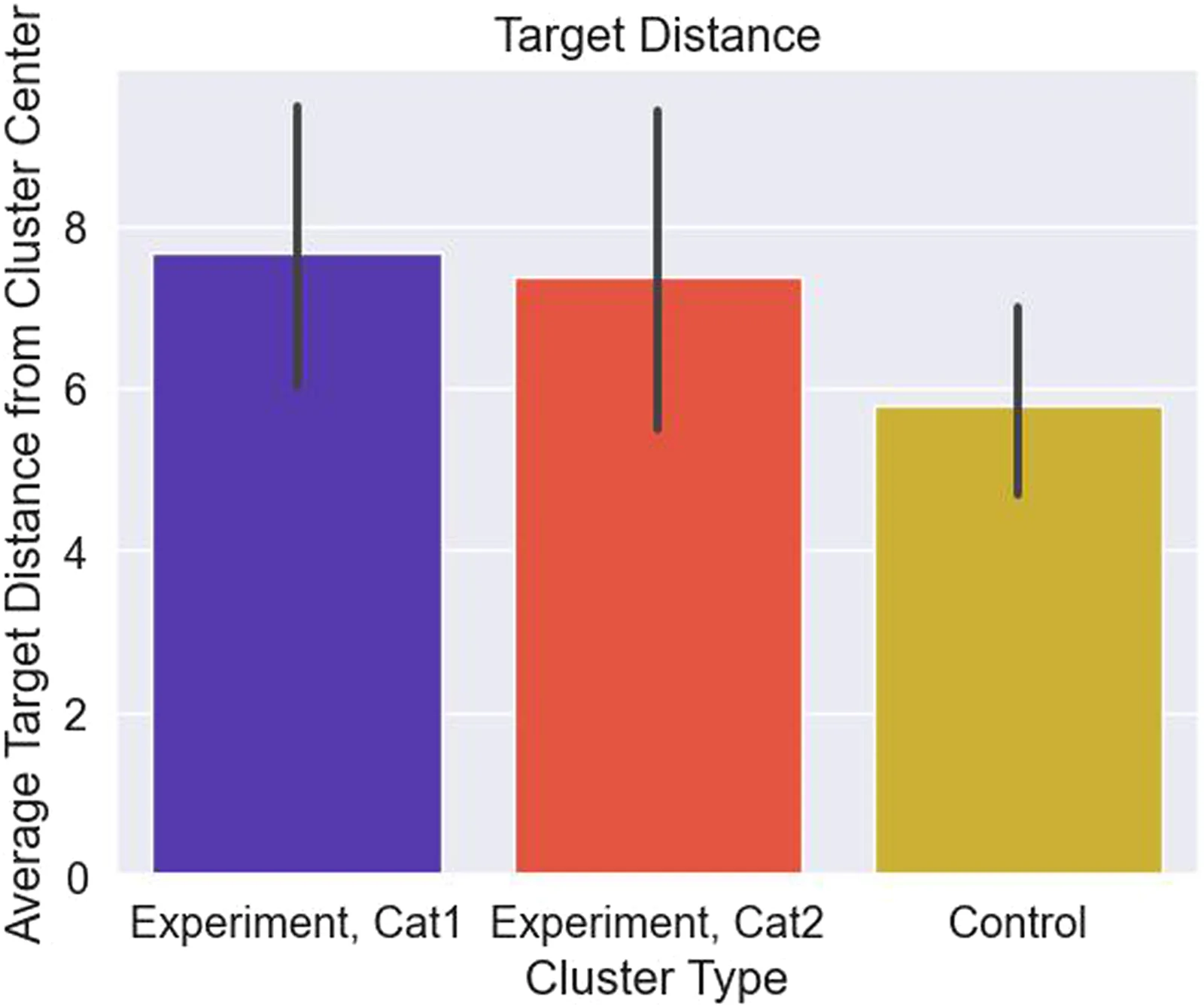
Target distance from center of cluster for a given category.

**Figure 5.**
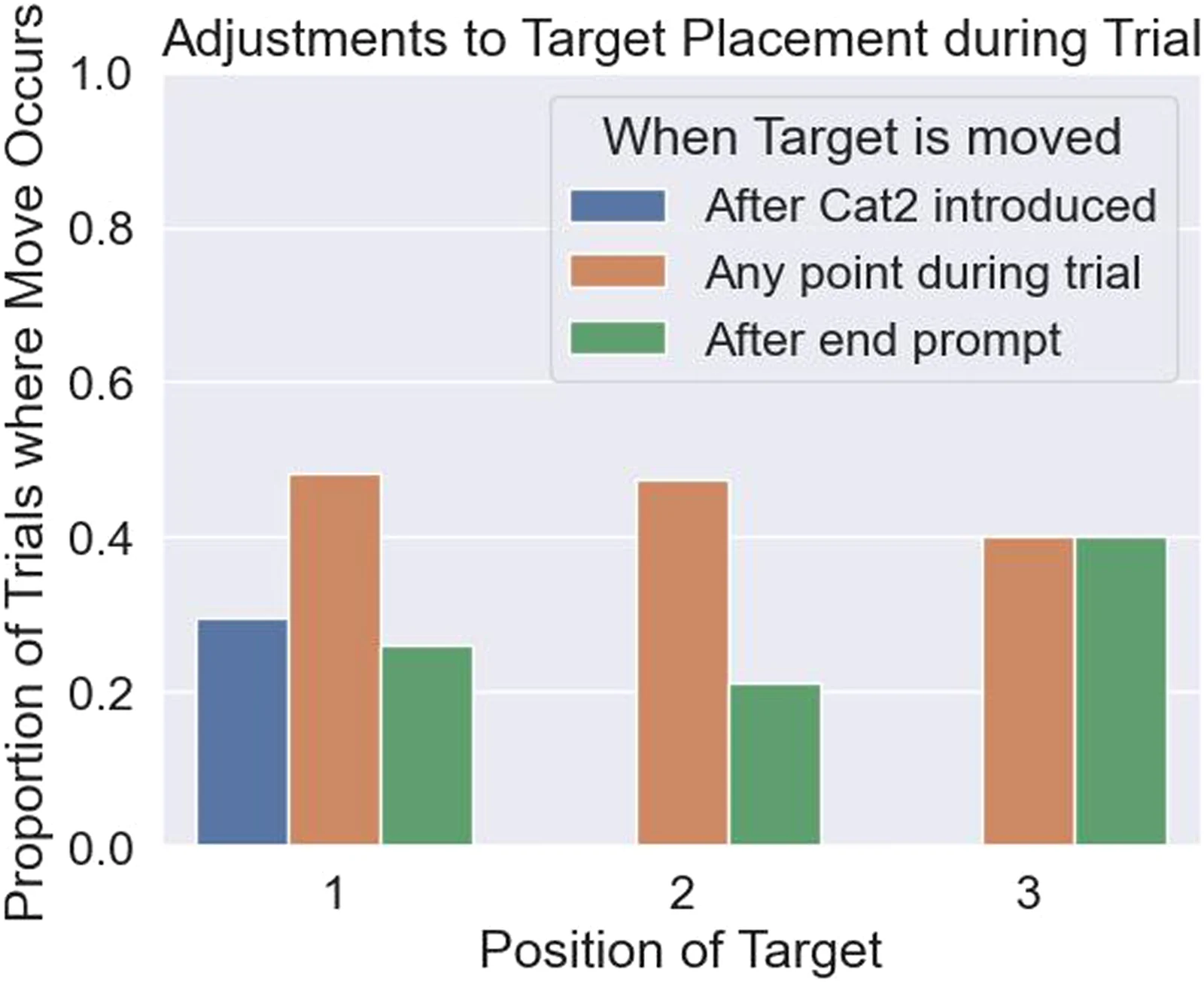
Movements of Target during experimental trials.

The data presented here show that GRIS can provide interesting insights into how participants make use of space while a trial is being conducted. They also demonstrate how GRIS experiments can be designed to control the order in which items are presented to be placed on the canvas, which is useful for controlling experiment variables. By gaining insight into how participants continually update spatial representations during a trial, researchers can obtain a clearer picture of real-time updates in response to new context, rather than relying on a static coordinate representation of the end of a trial.

### Interim Summary

In summary, GRIS is a simple, yet flexible, experimental paradigm that can accommodate a wide variety of research questions and designs. To validate GRIS’ effectiveness and demonstrate the kinds of analyses that it permits, we present a series of three experiments which capitalize on many of the features offered by the paradigm.

## EXPERIMENT 1: MULTI-DIMENSIONAL SIMILARITY

For decades, cognitive scientists have studied how people recognize patterns across a variety of cognitive domains (Chater & Vitányi, 2003; Edelman, 1999; Edelman & Duvdevani-Bar, 1997; Reed, 1972). In this experiment, we contribute to this literature by examining how one form of pattern recognition—similarity assessments—arises during language processing.

Prior work suggests that the cognitive sources of similarity are a concept’s familiarity (strength in memory), association (relationships with other concepts), and inherent perceptual likeness (surface appearance); see Hiatt and Trafton (2017) for an overview. Linguistic similarity, broadly defined, has also been shown to influence pattern recognition. For example, semantic similarity is well-known to produce priming effects (McNamara, 2005; Neely et al., 1989; Shelton & Martin, 1992), and syntactic similarity has shown similar effects (Lester et al., 2017; Reitter et al., 2011). Orthographic similarity improves recall accuracy in a probed serial-recall task (Lin et al., 2015), and phonological similarity has been shown to facilitate the learning of novel words (Papagno & Vallar, 1992).

While each of these features contributes to overall perception of similarity between linguistic units, how do people balance the multiple avenues of similarity to determine a single sense of similarity? Importantly, this research question would be difficult to test with standard paradigms, as it involves significant numbers of pairwise comparisons that would be both costly to run and difficult to interpret. Prior work in spatial arrangement paradigms has shown that people can use space to capture similarity judgments for objects within the same category that have slightly different features (i.e., bugs of different colors with varying amounts of legs) (Hout et al., 2013; Richie et al., 2020). Relatedly, previous research using the free classification task (i.e., Handel & Imai, 1972) suggests that people frequently sort objects according to a single dimension (Medin et al., 1987), though this pattern can be influenced by certain task distinctions (Milton et al., 2008; Regehr & Brooks, 1995). In this experiment, we demonstrate how the drag-and-drop functionality of spatial arrangement methods like GRIS captures similarity across many dimensions for multi-category similarity judgments, showing how certain types of similarity are more difficult to capture than others.

### Similarity Data

Materials for this experiment come from *Connections*, a free, publicly-available game hosted by *The New York Times*. In this game, players see a grid of 16 words and are told to separate the words into four distinct groups that are labeled; each item belongs to only one group. Importantly, each group of four words forms a labeled category, and these categories have varying difficulty assigned by the editors: yellow groups are the easiest, green groups the second-easiest, blue groups the second-hardest, and purple groups the most difficult.^13^ A full item and its corresponding solution are shown in Figure 6.

**Figure 6.**
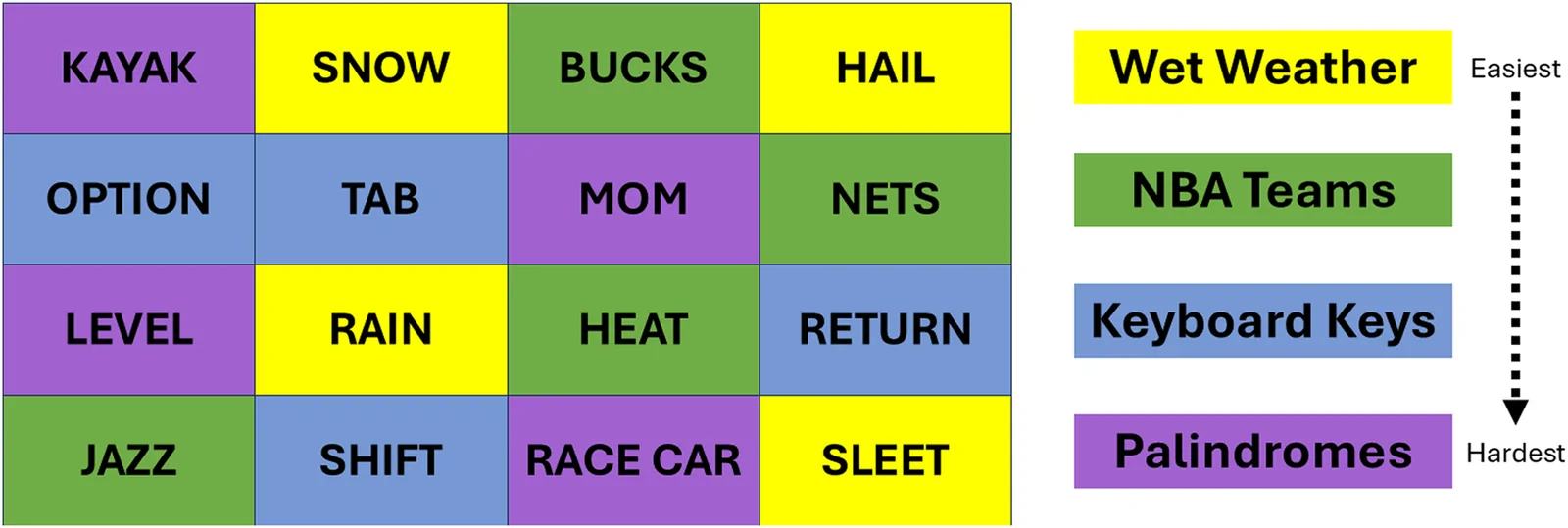
Sample *Connections* puzzle (left) with categories (right); puzzle in original format does not have colors. Colors reflect difficulty, as determined by the editors of the publication: yellow is the easiest, green is the second-easiest, blue is the second-hardest, and purple is the hardest.

*Connections* categories comprise different types of similarity groups. Following classifications from prior computational work on *Connections* (Samdarshi et al., 2024), two annotators categorized each group of words from 300 puzzles into one of three broader similarity categories: *Semantic Association* (from Figure 6, “wet weather”: hail, rain, sleet, snow), *World Knowledge* (e.g., “NBA teams”: bucks, heat, jazz, nets), and *Linguistic Reference* (e.g., “palindromes”: kayak, level, mom, race car); we present additional items in Table 2. As visualized in Figure 7, we see that the puzzle editors suggest that some similarities are considered more difficult than others: semantic association groups tend to occupy the easier categories, world knowledge groups tend to occupy the middle difficulties, and abstract linguistic reference groups tend to occupy the most challenging difficulties. However, it is unclear whether the assigned difficulty of the groups actually reflect the difficulty of the underlying cognitive processes required to find them.

**Table 2.** Example groups and their corresponding items for each of the three broad categories.

**Categories**	**Group**	**Items**
*Semantic Association*	Sports Professionals	coach, manager, player, scout
	Quick Peek	gander, glance, glimpse, look
*World Knowledge*	Rock & Roll Hall of Famers	heart, madonna, prince, queen
	Fictional Pirates	hook, jones, silver, sparrow
*Linguistic Reference*	Metric Prefixes	kilo, mega, micro, pico
	Homophones	peak, peek, peke, pique

**Figure 7.**
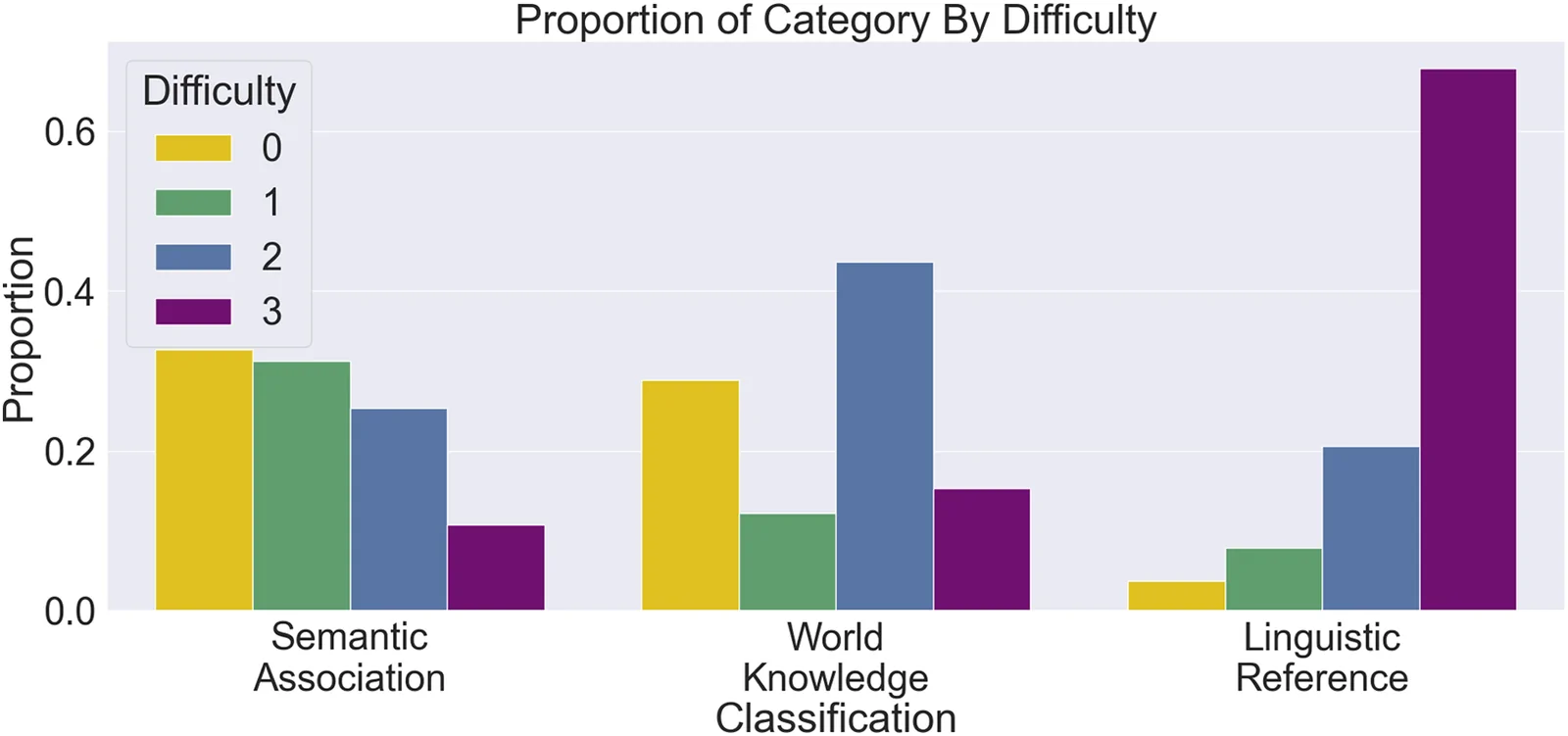
Distribution of similarity categories by difficulty. Difficulty levels closer to 0 are considered easier.

### Design and Procedure

#### Stimuli.

From our annotated data, we selected 10 puzzles that had at least two of the broad similarity categories. Given that we are using puzzles generated by the publication, we were unable to perfectly balance the different similarity categories across all puzzles.^14^

#### Procedure.

Participants saw a blank canvas with a word bank of words below. Participants were instructed to move these words onto the canvas according to how similar they were; similar words should be placed closer together. Participants were not instructed to make four groups of four, as they are required to in the actual game. Additionally, participants were instructed to use as much of the canvas as they felt was appropriate.

To train them on the task but to avoid biasing their decisions, participants completed two practice trials prior to the experiment where they grouped both shapes and numbers.

#### Participants.

Nineteen native speakers of English between the ages of 18 and 55 were recruited on Prolific.

### Results

For each trial, we collected incremental information for each move, along with the final position for all words. For every group within each trial, we computed two distance comparisons. within group distances were computed by calculating the average distance between every word in each group with other members of that same group. outside group distances were computed by calculating the average distance between every word within a group with every other word not in that group. We then computed by-participant *z*-scored distances.

Results are visualized in Figure 8. To determine how people used distance to group similar words together, we fit a linear mixed-effects regression model that predicted *z*-scored distance, with fixed effects of comparison (within group/outside group), category (semantic association/world experience/linguistic reference), and their full interactions, along with random intercepts for participants and items with random slopes for category.^15^

**Figure 8.**
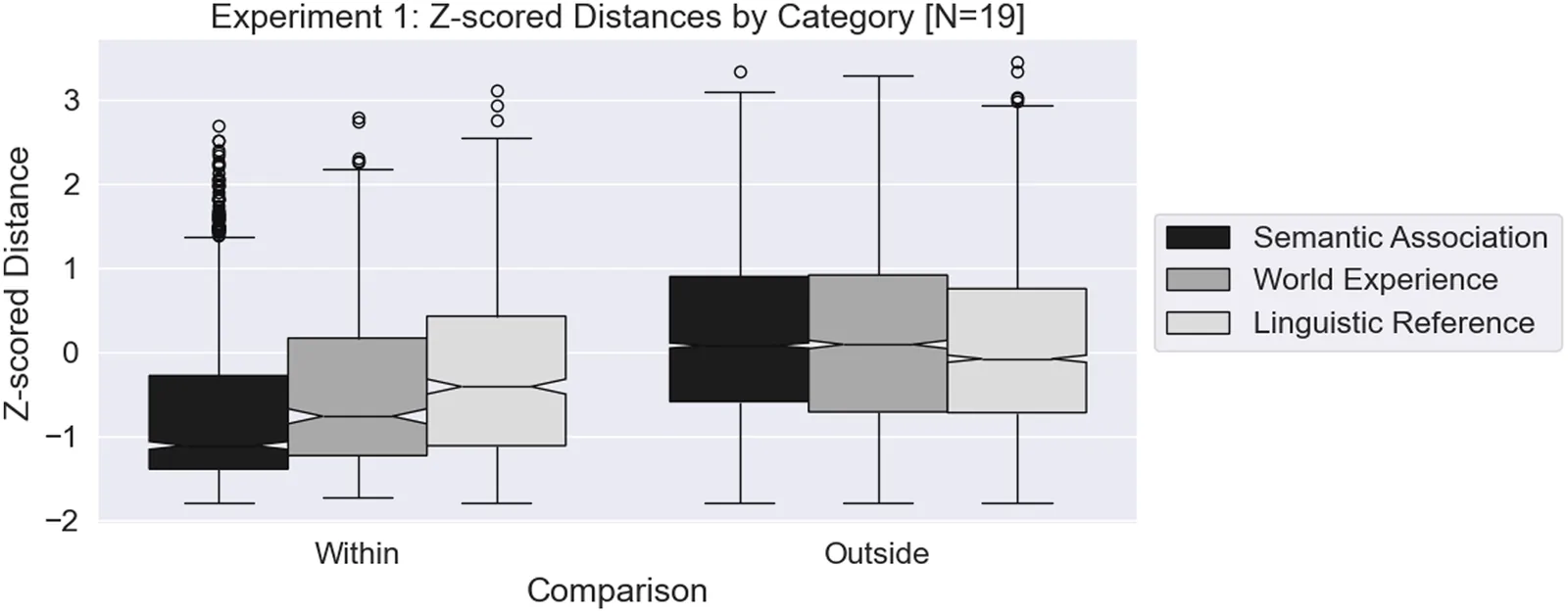
*Z*-scored distance by category for Experiment 1. Notches indicate bootstrapped 95% CIs.

Given the more complicated mixed-effects structure in comparison to our prior experiments, we report our model output in Table 3. We find a main effect of comparison such that outside group distances are significantly further apart than within group distances. within group comparisons reveal a category effect: semantic association clusters are closer together than both world experience and linguistic reference clusters. The significant interactions indicate that this category difference is attenuated for outside group comparisons: the gap between within group distances and outside group distances is significantly larger for semantic association groups than for either world experience or linguistic reference groups. These results indicate that category boundaries are sharpest for semantic association groups.

**Table 3.** Mixed-effect model output on all data from Experiment 1. **Bold** indicates significant effects.

**Effect**	$\hat{\beta }$	$\hat{\sigma }$	***t***	***p*-value**
(Intercept)	−0.711	0.028	−25.362	<0.001
**Linguistic Reference**	0.458	0.053	8.688	**<0.001**
**World Experience**	0.295	0.050	5.864	**<0.001**
**Outside**	0.867	0.029	29.682	**<0.001**
**Linguistic Reference : Outside**	−0.577	0.049	−11.741	**<0.001**
**World Experience : Outside**	−0.318	0.052	−6.110	**<0.001**

#### Similarity Strategies Across Participants.

When forming groups, people adopt various strategies for placing words on the canvas (see Figure 9). Some people, like participant B, form distinct clusters representing different groups; others, like participant A, intermix items more throughout the representational space.

**Figure 9.**
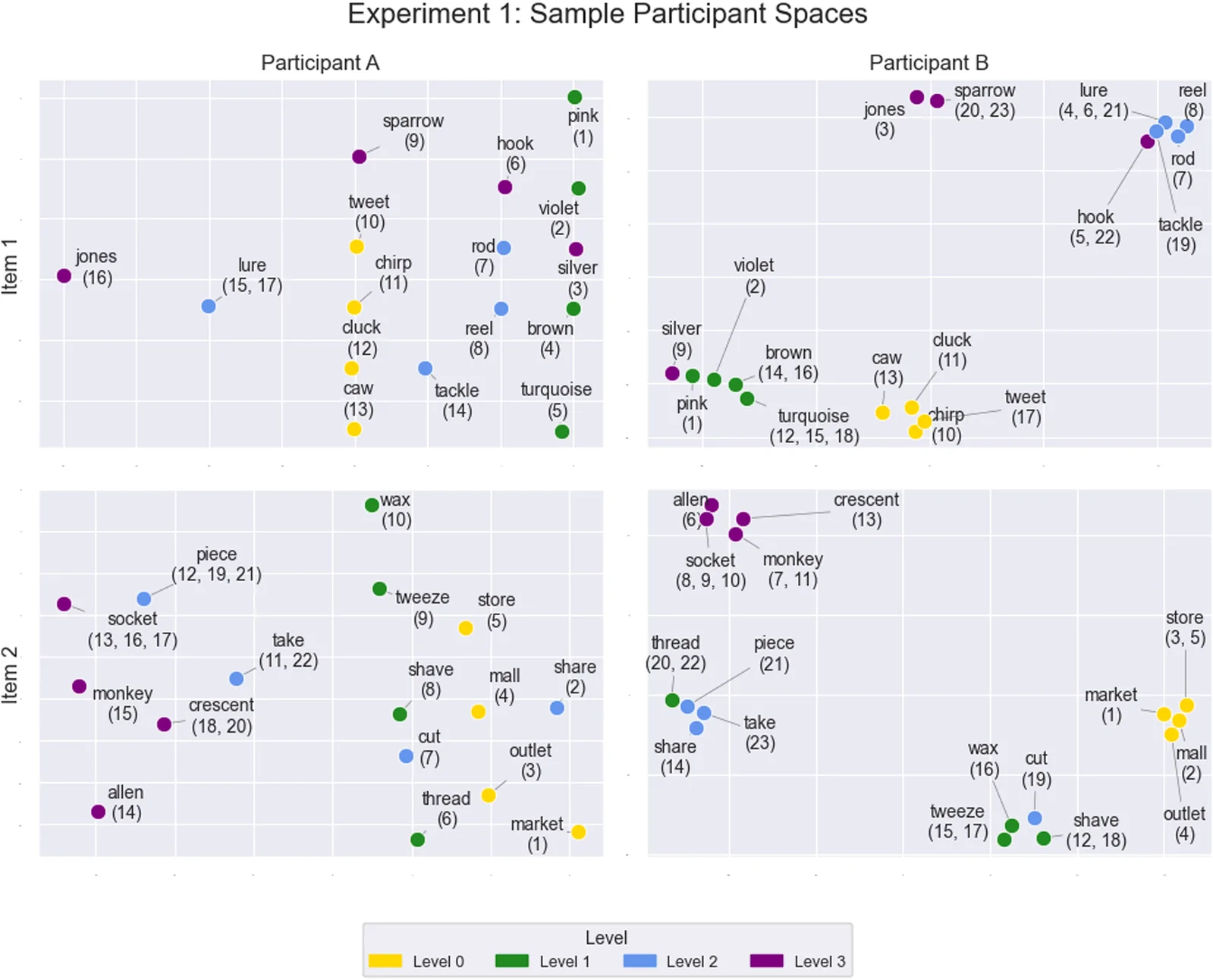
Representation spaces for two items (reconstructed through MDS). Number(s) indicate incremental move(s). Colors reflect difficulty level.

We also observe variations in how well participants are able to distinguish specific groups. For example, both participants A and B display some confusion between levels 1 and 2 in Item 2. Additionally, participant B fails to fully establish level 3 in Item 1. However, tracking the incremental placements of different words throughout the trial provides some insight into how participants construct categories. For example, in Item 1, the first and second words moved by both participants are “pink” and “violet,” respectively. While participant B goes on to place unrelated words belonging to other difficulty levels (“jones,” followed by “lure”), participant A continues by placing other color words nearby (“silver,” “brown,” “turquoise”). In Item 2, participant B is quick to distinguish the level 0 category: they place all four words belonging to that difficulty level onto the canvas first, clustered tightly together. In general, words belonging to “easier” difficulty levels (0 or 1) are placed earlier than those belonging to “harder” difficulty levels (2 or 3). By observing the order in which participants place words onto the canvas, we can see how categories emerge in their representational spaces.

### Discussion

In this experiment, we showed that certain similarity patterns are easier to find than others. More specifically, this experiment showed that semantic association leads to groups that are both closer together and also more isolated from other words, suggesting that these groups were easiest to find. These findings may derive from the fact that semantic association requires less reasoning to identify possible clusters of words, compared to other, more abstract groupings. We also showed that GRIS allows researchers to analyze the order in which items are placed onto the canvas, which provides insight into how people form groups as they progress through the trials: high-level patterns emerge in the aggregate, but we can still analyze how low-level variations surface.^16^

Beyond these results, we argue that the drag-and-drop paradigm of GRIS-based experiments works well to investigate the complex relationships between representations and reasoning: other paradigms—including rating tasks, forced-choice tasks, and priming tasks—would require significantly more opaque pairwise comparisons to accomplish the results of this study.

## EXPERIMENT 2: CATEGORY TYPICALITY

Category typicality assesses how “typical” an object is within a broader category (Farmer et al., 2006; Rosch, 1975). For example, “robins” and “sparrows” are found to be more typical representations of birds than “toucans” and “penguins” across cognitive domains, including language (Meints et al., 1999; Rosch, 1975) and vision (Maxfield et al., 2014). Traditionally, category typicality has been measured using rating or decision tasks (Rosch, 1975), production tasks (Rosch et al., 1976), or inductive-reasoning tasks (Osherson et al., 1990), all of which ask the participant to consider a specific word in relation to the broader category label. Recent computational work also suggests that computational models of language may learn some aspects of category typicality from the statistical usage distributions of everyday language (Misra et al., 2021), though these analyses focus on probability estimates from pre-trained language models rather than representational analyses.

In this experiment, we build a typicality-rating experiment using GRIS, finding that manipulating words in space both (1) replicates previous category typicality effects and (2) allows us to directly compare representational spaces between humans and models.

### Design and Procedure

#### Stimuli.

We used eight of the original 10 categories from Rosch (1975): *fruits*, *vehicles*, *weapons*, *vegetables*, *tools*, *birds*, *sports*, *clothing*. All items were in English. Each category has a list of approximately 50–60 words, where each word has a typicality rating that was averaged across 209 subjects; we use these ratings as our ground truth. To test whether the presence of different words modified typicality ratings, we constructed eight items that used ten words from each category; we did not use all of the words from Rosch (1975), as there would be too many words for participants to move on the screen.

#### Procedure.

A sample item for Experiment 2 is visualized in Figure 10. Participants saw a canvas with a word bank below. In the middle of the canvas was a bolded category label (i.e., **SPORTS**). Participants were told to move words from the bank onto the canvas according to how “typical” an example the word was of the category: words that were more typical examples of the category should be placed closer to the category label.

**Figure 10.**
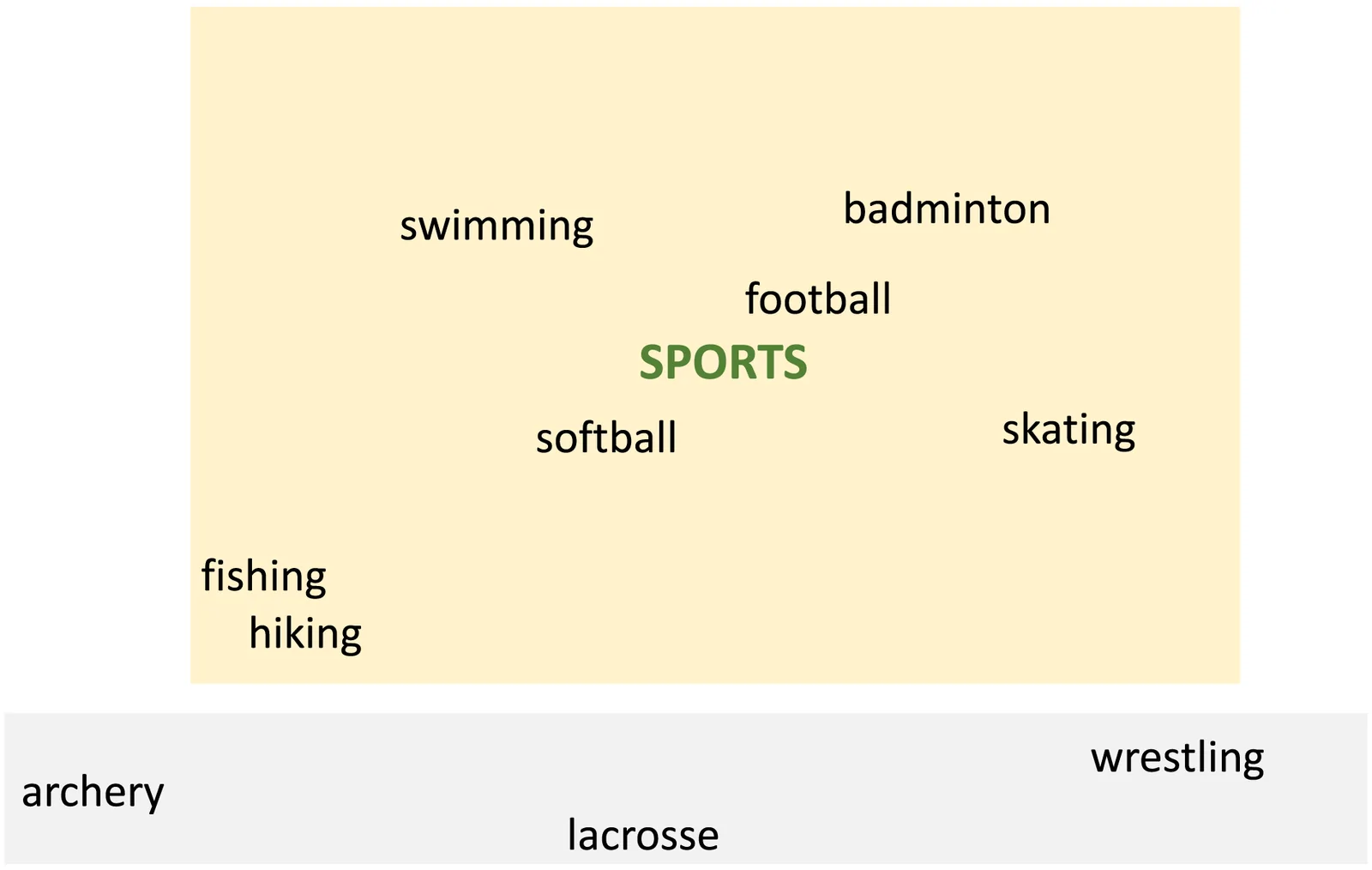
Sample trial for Experiment 2 (Typicality); font size enlarged to improve figure readability. Category label is marked in the center in green.

#### Participants.

Twenty-five participants were recruited using the online research platform Prolific. Participants were all native English speakers between the ages of 18 and 55.

### Results

As in Experiment 1, we collected the final positions for all words once the trial was complete. For each trial, we calculated every word’s distance from the center; we *z*-scored these distances by participant to ensure that all participants were comparable in how they used the space. Finally, following the rating averaging from Rosch (1975), we meaned the distances for each word across participants.

Experimental results are visualized in Figure 11. We find a strong correlation (*r* = 0.78) between the original rankings from Rosch (1975) and the distance of each word from its category label in our study, indicating that GRIS can be used to replicate prior category typicality results.

**Figure 11.**
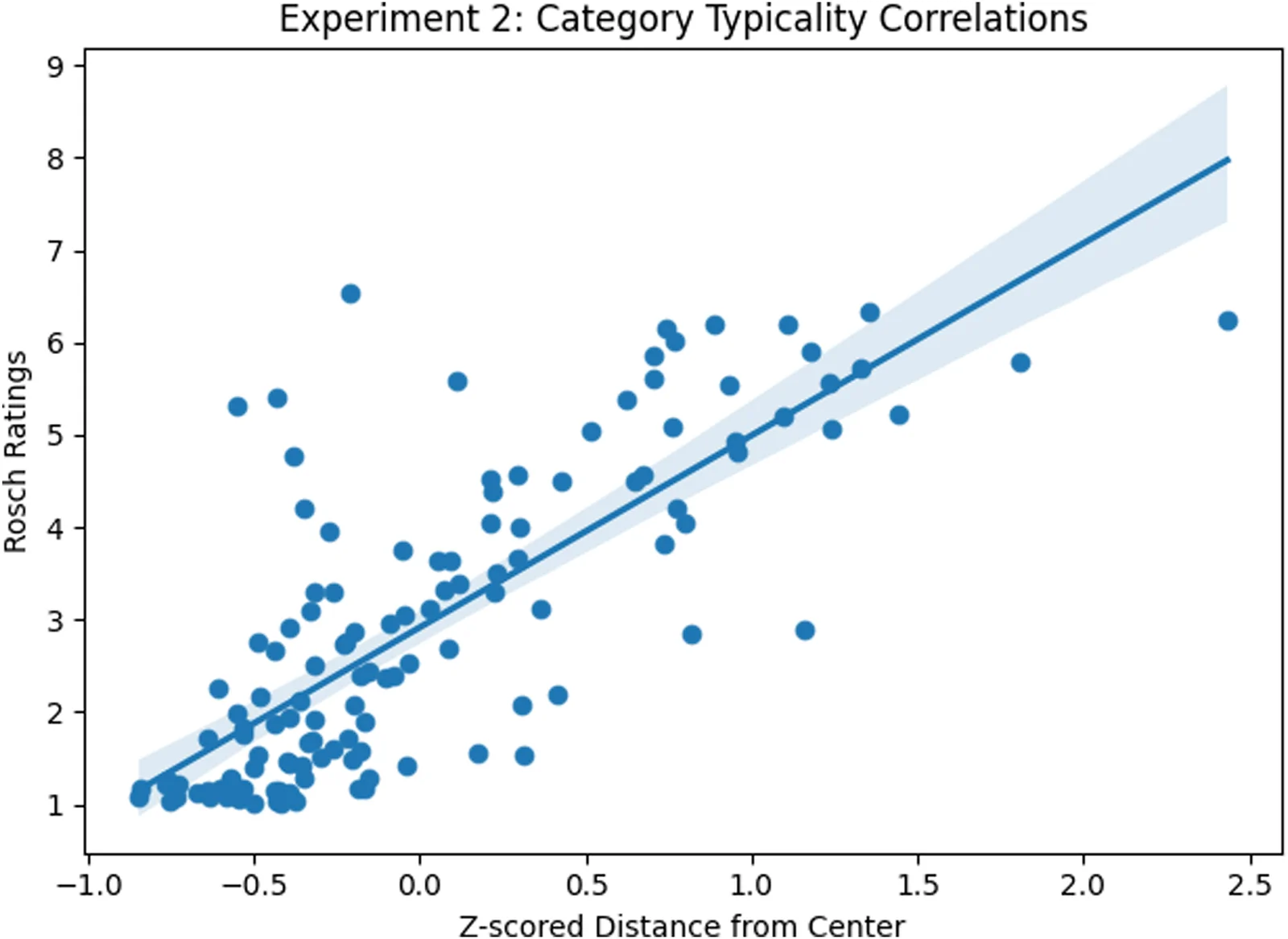
Correlation results for Experiment 2. *X*-axis indicates the *z*-scored distance from center for a word. *Z*-axis indicates the original ratings from Rosch (1975).

#### Analysis of Incremental Participant Data.

While we observe a strong correlation between the original rankings and our *z*-scored distance metrics (which are computed from each word’s final positions), we are also able to assess the strategies that people employ during the task: for example, participants could (1) sort the words according to how they encounter them in the task, (2) set the particular bounds of the typicality scale first, or (3) rank according to the scale. Accordingly, for each participant, we tracked the incremental moves for each object within each trial and its position from the center.

Correlation results for the incremental analysis are visualized in Figure 12. We find that participants generally put more typical items closer to the center earlier in the trial, then revise and place words further away as they progress. Broadly, these results suggest that people construct typicality assessments in a ranked fashion, where more typical exemplars of categories are prioritized over less typical exemplars.

**Figure 12.**
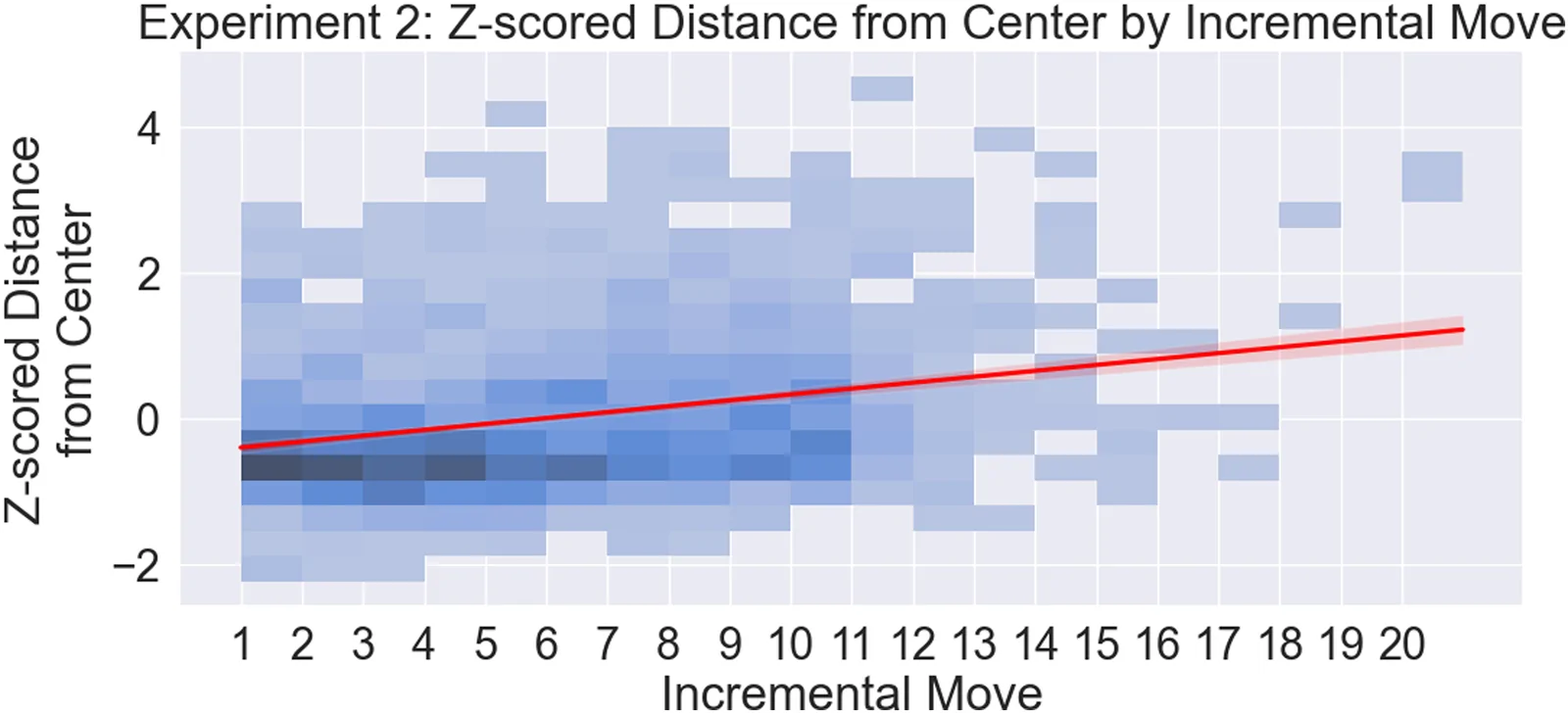
Correlation results between participants’ incremental movements of objects and their corresponding *z*-scored distance from the center. Darker colors indicate more movements in that bin.

#### Computational Analyses.

For our computational analyses, we extracted vector representations of words from three models: GLoVe 6B.300D (Pennington et al., 2014), BERT (Devlin et al., 2018), and GPT2 (Radford et al., 2019). For the non-contextual model (GLoVe), we gathered the raw vectors for both the word and the category label. Following Misra et al. (2021), for both of the contextual models (BERT & GPT2), we framed each word X with its category label Y using four sentence frames: *“A(n) X is a Y”* (Base), *“A(n) X is a typical Y”* (Typical), *“A typical Y is a(n) X”* (Typical Reverse), and *“A(n) X is considered to be a typical Y”* (Typical Verb Change); instead of gathering the probability of each word *X* and *Y* in the sentence, we extracted the vector representations of both the word and the label from each model’s final layers using the minicons Python package (Misra, 2022).^17^ Approaching our computational analyses in this way allows us to most directly compare the representational spaces constructed in the human experiment with those generated by computational models of language; our approach differs from that of Misra et al. (2021) in directly comparing model similarities to human similarity judgments rather than mapping model log-probabilities to human behavioral responses.

For each of the three models, we computed the Euclidean distance and the standardized cosine similarity between the vectors for every word and its corresponding category label.^18^ We then calculated the Spearman’s correlation for all possible model comparisons.

Results for these multiple-correlation analyses are visualized in Figure 13. We find that GRIS is the only set of representations that connect a word to its category label in a manner that strongly correlates with the original rankings from Rosch (1975); the distances between words and their labels for GLoVe representations only weakly correlate with the original Rosch rankings, though there is a slightly stronger correlation between GloVe distances and our experimental data. We note that representational distances in BERT and GPT2 correlate with one another, but fail to display any strong correlations with GLoVe or either set of experimental data; these differences are exacerbated if we use non-standardized cosine similarity correlation analyses. We also note that GRIS also has the highest average correlation coefficient across comparisons.

**Figure 13.**
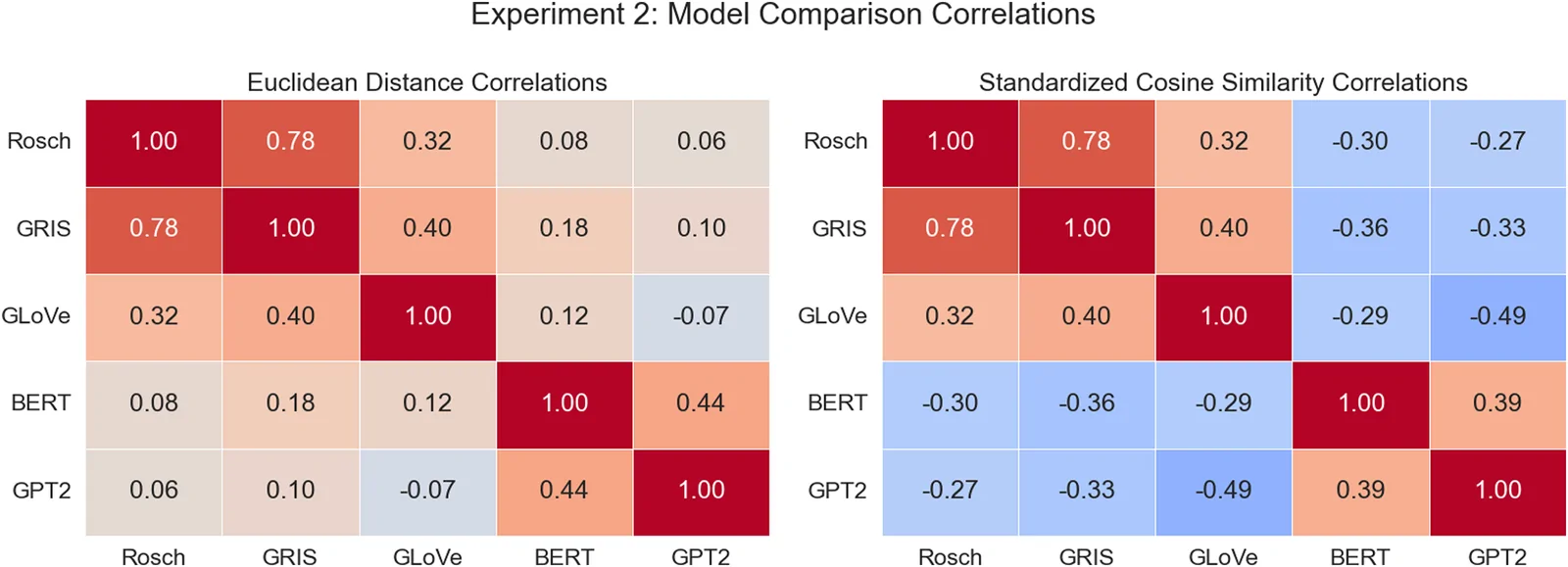
Correlation metrics between model representations and experimental results. Each cell corresponds to the Pearson’s correlation coefficient between the models and experimental measures on the *X*- and *Y*-axes. Red hues indicate greater similarity; blue hues indicate greater dissimilarity.

### Discussion

In this experiment, we replicated prior typicality representations for eight categories: we found that spatial arrangement methods like GRIS can capture well-established category typicality effects. We also found that people incrementally assess typicality from the “most typical” examples of a category to the “least typical” examples.

This experiment also demonstrated how GRIS constructs representational spaces more accurately than a number of well-established computational models. These findings differ from Misra et al. (2021), likely due to the fact that we conducted *representational* analyses and not *behavioral* ones: while previous computational work has shown that behavioral measures moderately align with human behavior, our work demonstrates that studies of human representations cannot simply rely on vectors generated by these models.^19^ In fact, the commonly-used cosine similarity metric is moderately *negatively* correlated with human behavior, so using language models as a similarity baseline in a human experiment could heavily distort the results.^20^ Instead, we can use efficient spatial arrangement methods of collecting data to quickly and easily collect similarity data in place of these models. It is also possible that GRIS results may serve as a useful bridge between representations in humans and computational models, as shown in the weak correlations between GRIS and BERT in our Euclidean distance analyses; we leave analyses of this topic for future work.

## EXPERIMENT 3: SENTENCE ACCEPTABILITY

Acceptability judgments probe what structures are (un)acceptable in a language: these structures can range from low-level judgments of phonological structure to high-level judgments of multi-sentence, multi-speaker discourses. In this section, we focus on sentence acceptability judgments in English.

For explanatory purposes, consider the sentences in (1)–(3). We adopt standard conventions for marking degrees of acceptability and grammaticality from linguistic research, where * indicates a sentence is ungrammatical, and # indicates a sentence is odd or slightly marked.

(1) *An girls is hungry.
(2) Randy wanted to write a novel.
(3) #Want to write, Randy did a novel.

While ungrammatical sentences like (1) are rated toward the boundaries of the acceptability spectrum, others display more gradient judgments: for example, sentence (2) is often preferred over sentence (3), even though both are grammatical sentences of English. Previous research primarily collects acceptability judgments using Likert scales (Gibson et al., 2011), forced-choice tasks (Mahowald et al., 2016), or response times (Konieczny, 2000). In isolation, such sentence acceptability judgments appear to be robust across experimental paradigms, suggesting that people have consistent preferences about the internal structure of their language (Sprouse, 2011; Sprouse et al., 2013). However, these measures do not always capture the relative relationship of sentence acceptability across structures. For example, people express consistent preferences: generally speaking, (2) > (3) > (1). But, each of these pairwise preferences reflects a different underlying scale: while (1) is less acceptable than (3) and (3) is less acceptable than (2), the former distinction is motivated by differences in grammaticality, while the latter distinction is motivated by differences in frequency and syntactic complexity. These differences suggest that grammaticality is unlikely to be totally binary, but rather a gradient comprised of multiple factors (Lau et al., 2017); others have interpeted these differences to mean that grammaticality and acceptability are distinct notions (see Sprouse (2018) for an overview).

Moreover, isolated syntactic judgments may also conflate degrees of acceptability: A rating of 3 for one construction may not be comparable to a rating of 3 for another construction, even though the ratings are identical, as the underlying mechanisms may differ. Capturing the contextual organization of syntactic acceptability across phenomena would help us understand the broader organization of human language understanding and cognition.

In this study, we use GRIS to replicate large-scale categorical sentence acceptability judgments from prior work, while also showing how the acceptability difference between sentence pairs can strongly vary depending on the context that they appear in.

### Design and Procedure

#### Stimuli.

All stimuli were drawn from Sprouse et al. (2013), which randomly sampled informal (i.e., not experimentally-tested) acceptability judgments of English sentence pairs from *Linguistic Inquiry*, a well-established journal in theoretical linguistics. After sampling these sentence pairs, Sprouse et al. (2013) collected acceptability ratings for each sentence within each pair to test whether the informal judgments were valid for larger populations; we will use these ratings to confirm that our findings correlate with prior work.

We sampled 72 pairs from the Sprouse et al. (2013) dataset. All 72 sentence pairs were classified according to the general linguistic phenomenon that their original paper tested; these classifications were drawn from the abstracts of the papers themselves. By labeling the linguistic phenomenon that each pair tests, we can then combine pairs of different classifications to understand how different syntactic phenomena influence sentence acceptability across structures, allowing us to obtain a broader understanding of the organizational preferences of acceptability judgments. Some sample classifications of phenomena are listed below in (4):

(4) a. Word Order:
    Fred mowed the green lawn. > *Fred mowed the lawn green.^21^
    b. Definites:
    This is a table. > *This is table.

From this set of 72 sentence pairs, we randomly selected 24 sentence pairs to serve as our target pairs: All participants saw each of these 24 sentence pairs. To test the impact of context on making these acceptability judgments, the remaining 48 items were broken into two sets of 24 sentences, each of which was paired with the 24 example items so that each target pair could appear in context with different phenomena. In sum, this process led to two sets of 24 items with four sentences (two pairs) each, where participants saw only one set.

#### Procedure.

See Figure 14 for a sample trial for Experiment 3. Participants saw four sentences below a gradiently-colored canvas, where the color gradient reflected a 5-point Likert scale.^22^ Participants were instructed to move the sentences from the bottom of the screen onto the canvas according to how “acceptable” the sentences were, according to their intuitions. Participants were told that the “most acceptable” sentences should be placed at the top of the canvas (interpreted as a 5 on a standard Likert scale), while the “least acceptable” sentences should be placed at the bottom (interpreted as a 1 on a standard Likert scale). They were also told that multiple sentences could occupy the same level on the scale.^23^ Sentence positions below the canvas were randomized for each item, and item order was randomized across participants.

**Figure 14.**
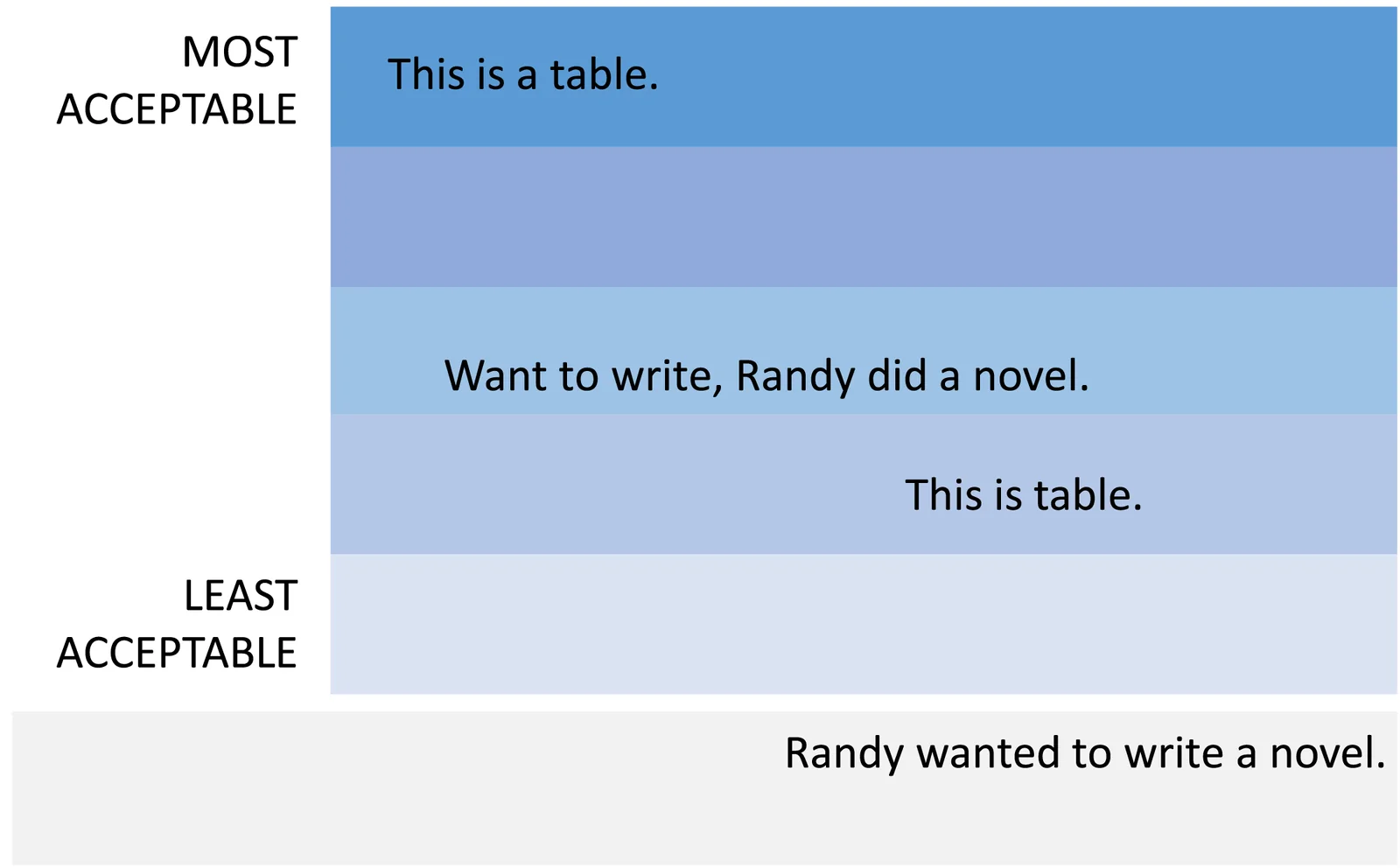
Sample trial for Experiment 3. Font has been enlarged for readability.

#### Participants.

Twenty-five participants were recruited using the online research platform Prolific. Participants were all native speakers of English between the ages of 18 and 55.

### Results

#### Base Acceptability.

To measure sentence acceptability judgments within each trial, we collected the final position of all sentences once the trial was complete. We *z*-scored acceptability ratings by participant to ensure that responses were compared on similar scales.

Results for Experiment 3 are visualized in Figure 15. To test whether unacceptable sentences were rated significantly lower than acceptable ones, we fit a linear mixed-effects model to the *z*-scored acceptability rating, with a fixed effect of sentence type (acceptable/unacceptable), and random intercepts for participants and items.^24^ Participants rated the unacceptable sentences as significantly less acceptable than the acceptable ones ($\hat{\beta }$ = −0.184, $\hat{\sigma }$ = 0.062, *t* = −29.70, *p* < 0.001); these sentence ratings also strongly correlate (*r* = 0.88) with those found by Sprouse et al. (2013).^25^

**Figure 15.**
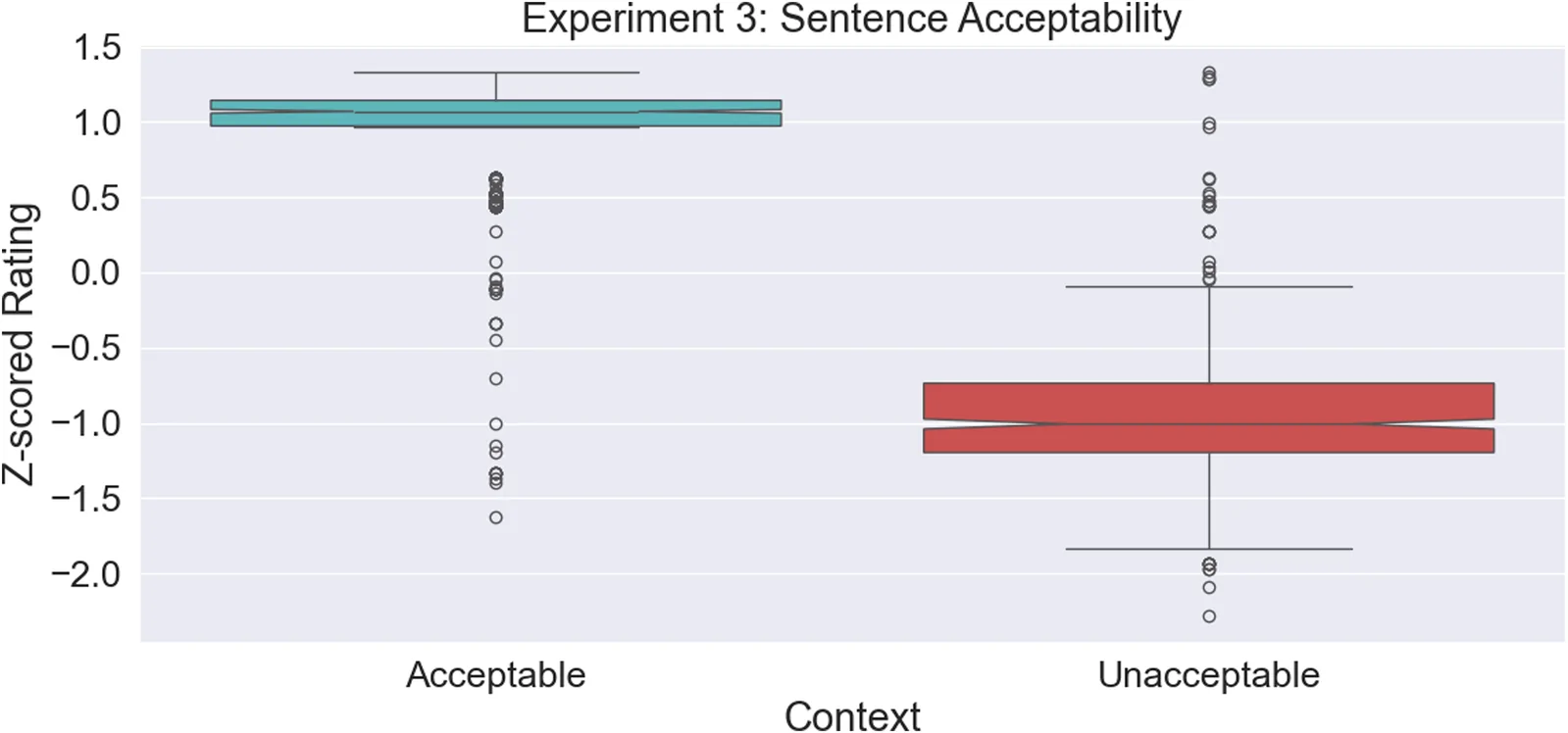
Base acceptability results for Experiment 3. Notches indicate 95% bootstrapped CIs.

#### Contextual Acceptability.

In addition to the basic acceptability analyses in the previous section, we measured how acceptability differences varied within each target pair according to the classification of the context pair that was present in the trial. To do so, we calculated the difference between each sentence in the target pair, then averaged the ratings within each context classification.

Results for contextual acceptability differences are shown in Figure 16. We find that some phenomena display similar levels of acceptability regardless of context (e.g., *Agreement*), while others show significant variation (e.g., *Word Order*, *Clause*). For example, consider the *Word Order* classification for the target pair from (4-a): *Fred mowed the green lawn* > *Fred mowed the lawn green*. When placed in the context of a sentence pair that modulates *Definites*, the difference between the *green lawn* and *lawn green* sentences was approximately 2.1 on a 5-point Likert scale; but, when placed in the context of a sentence pair that modulates *Objecthood*, the difference between the *green lawn* and *lawn green* sentences was approximately 3.1. From an experimental perspective, these varying differences have significant consequences on how researchers interpret acceptability judgments: A difference of ∼3 points on a 5-point Likert scale easily distinguishes an acceptable sentence (5) from an unacceptable one (2), whereas a difference of ∼2 points could be the distinction between a totally acceptable sentence (5) and a moderately acceptable one (3). From a cognitive perspective, these differences highlight possible avenues for furthering work on syntactic processing across construction types.

**Figure 16.**
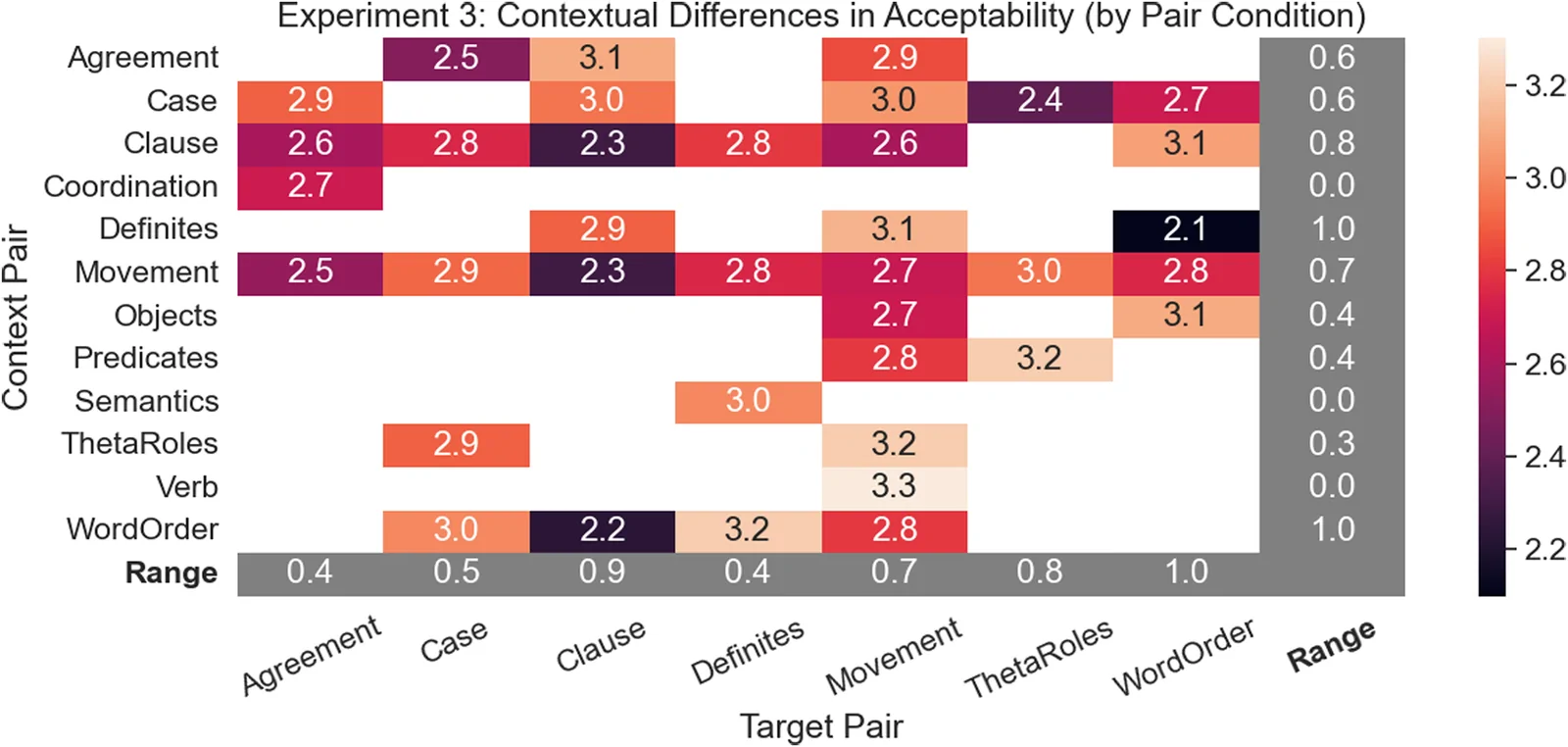
Contextual acceptability results for Experiment 3. *X*-axis represents the classification for the target pair. *Y*-axis represents the classification of the context pair. Cells indicate difference between acceptable and unacceptable sentences within each target pair; darker colors indicate smaller differences on a 5-point Likert scale. Ranges for columns and rows are in grey (bottom and right of the graph, respectively).

### Discussion

The results of this task show that GRIS can be used to reliably replicate prior experimental results involving pairwise comparisons: GRIS can be used to capture categorical differences between items that correlate with prior work. In the case of grammaticality, we find that grammatical sentences are rated significantly higher than ungrammatical sentences across contexts.

From a broader perspective, the results of this experiment support recent discussions about grammaticality and acceptability as distinct notions which are targeted during rating tasks: for example, previous work has found that participants are malleable to the type of judgments (either “grammatical” or “acceptable”) that are requested in the instructions (Langsford et al., 2019), or to repeated exposures of an item during an experiment (Zervakis & Mazuka, 2013). In our study, we find that the acceptability differences between grammatical and ungrammatical sentences in a sentence pair varies according to the experimental context of that pair. These different experimental contexts cause participants to use distinct scales acceptability scales, as presented qualitatively in examples (1)–(3). More plainly, we show how the same 1–5 acceptability scale can be used to reflect different underlying processes. Future work using GRIS can be used to create multiple arrangements of the same set of sentences, to more accurately target what factors influence scalar manipulation; this process would reflect a similar process to the multi-arrangement method used in Kriegeskorte and Mur (2012).

## GENERAL DISCUSSION

Like SpAM and other spatial arrangement paradigms, GRIS relies on natural human intuitions around space to build contextual and interpretable approximations of cognitive representations. We have used GRIS as a validation for how geometric arrangements can be used to approximate human representational spaces, allowing experimenters to model representational spaces both within class and across classes, while also providing information about the relative relationships between objects on the grid across participants.

Additionally, we have extended prior work in spatial arrangement paradigms (Goldstone, 1994; Hout et al., 2013; Kriegeskorte & Mur, 2012) to multiple cognitive phenomena. Recent work (highlighted in our case study) has used GRIS to demonstrate how people update their representation of categories according to the context in which the targets appear. In Experiment 1, we showed how people geometrically construct multi-class, multi-dimensional similarity relationships between 16 objects. In Experiment 2, we found that people assess typicality according to rank-order, where most typical objects are moved first. In Experiment 3, we illustrated how sentence acceptability judgments vary according to the structures around them, supporting prior work on the variable and gradient qualities of grammaticality distinctions.

From a computational perspective, GRIS and other spatial arrangement paradigms create multi-dimensional representations that are easily comparable to popular computational models of language, such as Large Language Models (LLMs). We showed that LLM semantic spaces currently do not map well onto human representational spaces, though GRIS could provide a tool for bridging that divide: GRIS-informed representations can be used to further explore mismatches between humans and models to help understand what aspects of human cognition are not determinable from co-occurrence statistics alone.

Finally, throughout our experiments, we have shown how low-level, incremental data about object movements can provide further nuance to experimental results across these domains.^26^ GRIS collects other forms of information, such as specific timing data for each movement, which may also shed light into important cognitive processes. More broadly, the incremental data that GRIS collects may be used to address other cognitive questions that we have not covered in this paper: For example, how do individuals vary in how they construct their representation spaces? How does incremental timing data reflect the complexity of the cognitive processes underlying participant decisions? We hope that future research using GRIS may provide answers to questions like these.

Given the flexibility of the tool and the established toolkit to build, run, and analyze GRIS experiments, we hope that other researchers will use GRIS to expand our understanding of cognition. For example, future work using GRIS could compare representation spaces across and within modalities (e.g., text, image, audio) to evaluate how multi-modal representations align. Moreover, prior spatial arrangement studies have been validated with both small sample sizes (*N* < 10) and large ones (*N* > 150) (Kriegeskorte & Mur, 2012; Richie et al., 2020); we hope that future work conducts large-scale studies using GRIS to further validate the contribution of incremental information. While we demonstrate how incremental information is cognitively informative despite the relatively small sample sizes across our three experiments, larger participant pools would allow for more robust and quantitative analyses of the processes and strategies that people use when structuring their representation spaces.

In conclusion, we note the centrality of spatial reasoning and language to cognition, and how unifying them can 1) make an experiment more intuitive, 2) yield more holistic and contextually-relevant results, and 3) construct representations that facilitate comparisons between humans and computational models.

## ACKNOWLEDGMENTS

Parts of this work were reported in the Proceedings of the 47th Annual Meeting of the Cognitive Science Society; we thank the attendees of the 47th Annual Meeting of the Cognitive Science Society for their helpful feedback. We would also like to thank members of the Cornell Linguistic Meaning (LiMe) Lab and the Computational Psycholinguistics Discussions Group (C.Psyd) for their comments on this work during its development. Finally, we thank Ted Gibson, Kyle Mahowald, and the anonymous reviewers for their feedback on earlier versions of this article.

## FUNDING INFORMATION

This work was funded by a research fund through the Cognitive Science program at Cornell University.

## AUTHOR CONTRIBUTIONS

J.R.S.: Conceptualization; Data curation; Formal analysis; Funding acquisition; Investigation; Methodology; Project administration; Software; Validation; Visualization; Writing – original draft; Writing – review & editing. A.W.: Data curation; Formal analysis; Investigation; Methodology; Visualization; Writing – original draft; Writing – review & editing. M.v.S.: Conceptualization; Methodology; Project administration; Supervision; Writing – original draft; Writing – review & editing.

## DATA AVAILABILITY STATEMENT

The items, data, and analysis code for Experiments 1–3 can be found at the following link: https://osf.io/94gck/view_only=1ed03a3757fe44ba9a036510be60b7c6.

## Notes

^1^ The items, data, and analysis code for Experiments 1–3 can be found at the following link: https://osf.io/94gck/?view_only=1ed03a3757fe44ba9a036510be60b7c6.^2^ In this paper, we focus on *linguistic* representations, though GRIS can be easily extended to approximate other kinds of cognitive structures and relationships such as in vision or acoustics.^3^ As will be described later in this article, GRIS is not designed to capture on-line processing. Accordingly, we do not elaborate on the use of space in psycholinguistic tasks such as self-paced reading (Just et al., 1982) or Maze (Forster et al., 2009).^4^ Mathematically, for *n* items, one would need to collect *n*(*n* − 1)/2 judgments.^5^ Given the many variables that affect human thought, it is possible that these inconsistencies (at least partially) reflect important cognitive processes. For example, Mussweiler (2002) found that anchoring effects (i.e., recent items affecting judgments of later material) were strongly influenced by whether participants had been asked to focus on similarities or differences. Accordingly, spatial arrangement methods collected through GRIS may reflect one view of the representation space, though these representations may differ from judgments collected through other methods.^6^ We use “proximity” as a catch-all term for similarity in the vector space, given that there are a variety of metrics—Euclidean distance, cosine similarity, Spearman’s *ρ*, etc.—to determine representational similarity.^7^ See Apidianaki (2023) for an overview.^8^ Some research in activation & representation engineering (e.g., Turner et al., 2024; Wu et al., 2024) demonstrates how these representations can be fine-tuned to perform better on down-stream tasks; we do not discuss these approaches in detail, though we do address them in the discussion.^9^ We do not impose an upper limit on the number of objects that a participant can meaningfully manipulate. Participants in our studies saw a maximum of 16 objects at once (as will be discussed in Experiment 1); prior work has used 20 objects (Goldstone, 1994) and 27 objects (Hout et al., 2013), suggesting that participants may be able to move many objects simultaneously. However, the number of objects that a participant sees is contingent on both the research question and the modality of the objects.^10^ The GRIS toolkit is publicly available on GitHub at the following link: https://github.com/johnstarr-ling/gris-toolkit. Currently, GRIS experiments are run on PC Ibex (Zehr & Schwarz, 2018), a free, online research platform intended for experiments in psycholinguistics and cognitive science.^11^ The data presented here are a small subset of data from other work that is currently under review, but which highlights some of the strengths of GRIS and whose results would be difficult to obtain under other methods.^12^ The complete list of control trials used for this subset of data is presented in Appendix B.^13^ We note that all categories (and their corresponding difficulties) are suggested by the puzzle editors at *The New York Times*; while we share the editors’ intuitions that some kinds of groups are more difficult to find than others, we are not simply evaluating whether the editors are assigning the appropriate difficulty to different types of groups. Instead, we are probing the cognitive question surrounding multi-class similarity comparisons.^14^ Instead, categories were balanced to be approximately 40% semantic association, 30% world knowledge, and 30% linguistic reference.^15^ The complete model formula was: Distance ∼ Comparison * Category + (1 + Category | item) + (1 + Category | participant); this is the maximal model that reaches convergence. The baseline conditions were the within group and semantic association groups, respectively.^16^ We note that that these low-level variations may also show differences in how people use similarity “locally” (or for objects near each other) compared to how they use similarity “at a distance” (or for objects that are dissimilar from each other).^17^ Previous work has suggested that intermediate layers may encode more abstract relations and concepts compared to the final output layer (Jawahar et al., 2019; Sajjad et al., 2022). Accordingly, we replicated our analyses in this section using an intermediate layer (layer 6 of 12) for both models; results can be found in Appendix C.^18^ To avoid issues with *anisotropy* during cosine similarity calculations, we followed the vector standardization procedure recommended by Timkey and van Schijndel (2021).^19^ We acknowledge that we are comparing a behavioral measure to an internal representation of a language model (as has been done before), where a more accurate comaprison would be to compare model representations with neuroimaging data (i.e., Schrimpf et al., 2021).^20^ As noted by one reviewer, it is possible that only some of the information in the word embeddings provides the meaningful comparison in a typicality study such as this one. As such, perhaps a *subspace* of the multi-dimensional space may correlate better with the human data. To overcome this limitation, some prior work has proposed alternative representational analyses: for example, feature-reweighted representational similarity analyses allows certain dimensions to contribute more to similarity metrics (Kaniuth & Hebart, 2022), and projection analyses which orthogonally projects word embeddings onto particular feature subspaces (Grand et al., 2018). We hope that future work using GRIS can demonstrate how these methods can be used to both a) align computational representations with GRIS networks, and b) analyze the properties of GRIS networks themselves.^21^ While the example provided here does introduce a resultative construction, the primary arguments of the original paper discuss the construction’s implications on word order.^22^ In most psycholinguistic studies involving minimal pairs, participants typically see only one item from the pair. Given we are interested in how sentence acceptability differences vary in the context of different constructions, we differ from these prior designs. However, we follow Marty et al. (2020), which suggests that joint presentation of both items in a sentence pair leads to more sensitive acceptability responses.^23^ Participants were allowed to place multiple sentences within each scale level so that acceptability differences within each scale item could still be captured (i.e., a strong 3 could be distinguished from a soft 3).^24^ The complete model formula was: *Z*-scored Rating ∼ Type + (1 | item) + (1 | participant). The baseline was the “Acceptable” condition.^25^ One reviewer notes that this strong correlation may be driven by the differences between grammatical and ungrammatical conditions. We present separate analyses of these data in Appendix D.^26^ We note that incremental positional data for objects may sometimes be redundant with their final positions. However, such redundancy may still be cognitively informative: For example, objects that have not been moved (or have only been minimally moved) may reflect a participant’s confidence in the object’s relation to other objects.

## Supplementary Material


