# Supplementary material for: Approximating Cognitive Representations Using Space
Source: Open Mind (Camb). 2026 Oct 3;10:1567–92. doi: 10.1162/OPMI.a.389 (PMC13733553; doi:10.1162/OPMI.a.389)
Supplement: Supplementary file 1 [file opmi-10-1567-s001.pdf]

A: APPENDIX A

Post-Hoc Statistical Analysis of WITHIN ***group Distances by Difficulty***

In this appendix, we present a post-hoc statistical analysis of WITHIN group distances by difficulty. This analysis is motivated by the idea of understand how puzzle difficulty, as suggested by the New York Times, may affect WITHIN group distances.

To conduct this analysis, we looked at the WITHIN group subset of our data. We reapplied the by-participant z-score transform, as the outside distances would unnecessarily modulate the magnitude of the differences that participants created within groups. We ran a mixed-effects model that predicted the z-scored distance, with fixed effects of CATEGORY, PUZZLE DIFFICULTY, and their full interaction, along with random intercepts for item and participant with by-CATEGORY random slopes.[1]

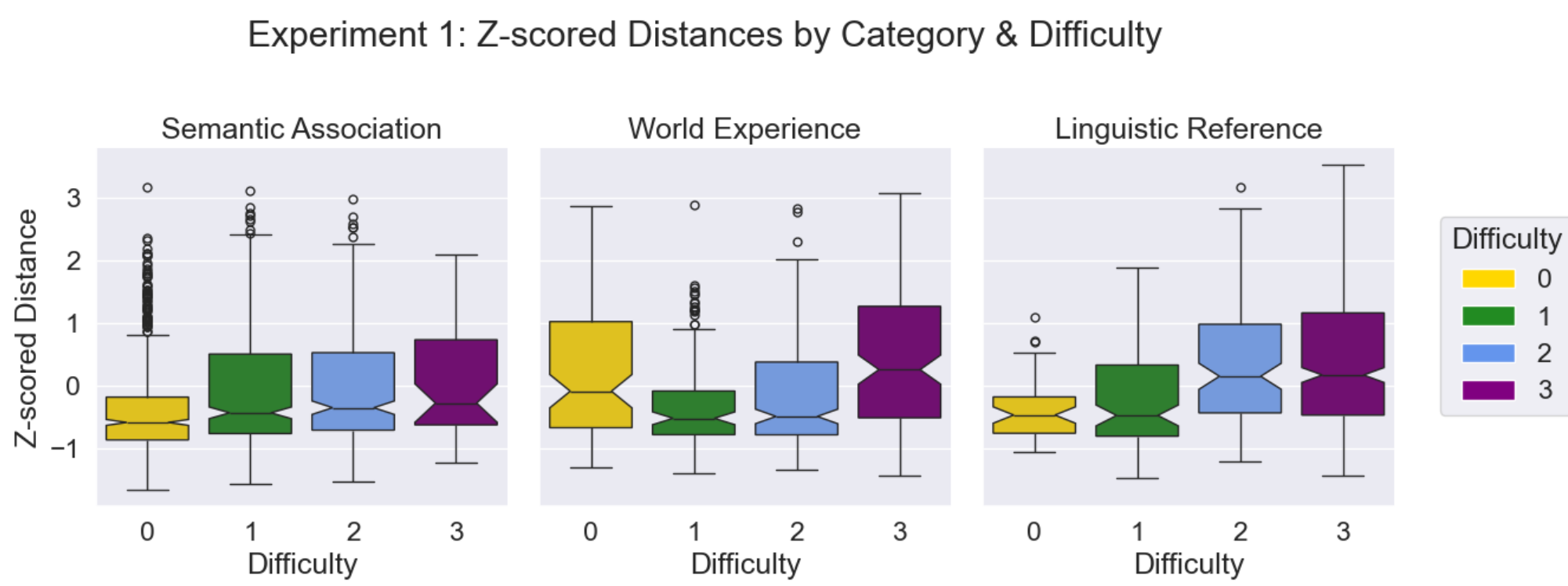

Figure A.1. Z-scored distance by category and difficulty for post-hoc analysis for Experiment 3 data. Notches indicate bootstrapped 95% CIs.

Model outputs are reported in Table A.1; results are visualized in Figure A.1. We find that, as expected, puzzle distances become further apart with each difficulty level, suggesting that higher difficulty groups are more difficult to determine. We also find some significant interactions: for example, we find that the WORLD EXPERIENCE category groups are significantly more distant from each other, suggesting that this category is challenging when it is considered the easiest difficulty. Broadly, these results reflect the idea that some kinds of similarity are more difficult than others, even if they have the same assigned puzzle difficulty.

[1] The complete model formula was: DISTANCE $\sim$ CATEGORY*PUZZLE DIFFICULTY + (1 + CATEGORY | item) + (1 + CATEGORY | participant); this is the maximal model that reaches convergence. The baseline conditions were the SEMANTIC ASSOCIATION group and difficulty 0, respectively.

**Effect**	$\hat{\beta}$	$\hat{\sigma}$	t	p **-value**
(Intercept)	-0.820	0.010	-8.298	$<$0.001
Linguistic Reference	-0.267	0.304	-0.878	0.407
**World Experience**	1.118	0.230	4.860	$<$**0.001**
**Level 1**	0.160	0.067	2.369	**0.018**
**Level 2**	0.192	0.073	2.642	$<$**0.01**
**Level 3**	0.420	0.155	2.707	$<$**0.001**
Linguistic Reference : Level 1	0.283	0.325	0.871	0.411
**World Experience : Level 1**	-1.614	0.245	-6.456	$<$**0.001**
**Linguistic Reference : Level 2**	0.789	0.323	2.445	**0.041**
**World Experience : Level 2**	-0.876	0.1415	-6.189	$<$**0.001**
Linguistic Reference : Level 3	0.532	0.340	1.569	0.149
**World Experience : Level 3**	-1.013	0.251	-4.037	$<$**0.001**

Table A.1. Mixed-effects output on post-hoc WITHIN group distance comparisons. Bold indicates significant effects.

B: APPENDIX B

Control Trials for Case Study

In this appendix, we present the control trials for the Case Study in Section 3.

Target	Related Category	Unrelated Category
bank	RIVER FEATURE:<br>waterfall, rapids, floodplain	COLOR:<br>green, blue, pink
bank	RIVER FEATURE:<br>waterfall, rapids, floodplain	UNIT OF TIME:<br>hour, minute, day
bank	INSTITUTION:<br>post office, hospital, library	PART OF HAND:<br>finger, knuckle, wrist
plant	BIOTA:<br>bacteria, fungus, animal	FURNITURE:<br>table, bed, dresser
plant	BIOTA:<br>bacteria, fungus, animal	UNIT OF DISTANCE:<br>mile, foot, inch
plant	MANUFACTORY:<br>factory, mill, forge	OFFICIAL PERSON:<br>director, chief, executive

Table B.1. Full suite of control trials for the case study showing that people update polysemous word representations.

C: APPENDIX C

In this appendix, we repeat our computational analyses from Experiment 2 using representations from an intermediate layer (layer 6) for both GPT2 and BERT. Results are visualized in Figure C.1.

Generally, our results from Experiment 2 maintain: GRIS distances strongly correlate with the human ratings, and also (at least weakly) correlate with the metrics computed from computational models. However, we note a few distinctions. First, Euclidean distance calculations for representations from intermediate layers from BERT more strongly correlate with human ratings, GLoVe distances, and GRIS ratings than such calculations for representations from the final layers; we do not see a significant change for GPT2. Second, cosine similarities from BERT and GPT2 become significantly less correlated with all other metrics when the representations come from intermediate layers.

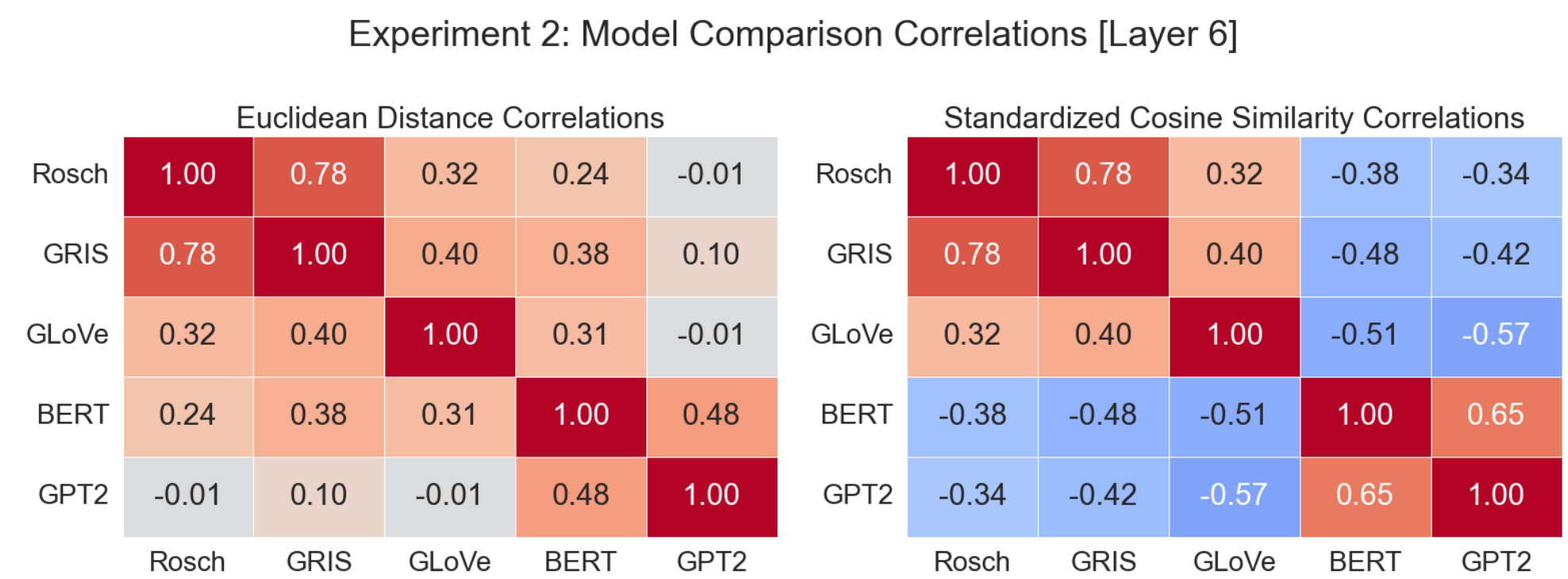

Figure C.1. Correlation metrics between model representations and experimental results. Each cell corresponds to the Pearson's correlation coefficient between the models and experimental measures on the X- and Y-axes. Red hues indicate greater similarity; blue hues indicate greater dissimilarity. All representations for GPT2 and BERT were extracted from Layer 6.

D: APPENDIX D

In Experiment 3, we found that differences in GRIS positions between sentences strongly correlated to the original ratings from Sprouse, Schütze, and Almeida (2013). In this appendix, we test whether these results are driven by the differences in acceptability by splitting the data into two subsets (acceptable and unacceptable) and seeing how our GRIS measures correlate with the original ratings.

Broadly, correlations weaken when split into different grammaticality conditions: we find a weak correlation for acceptable sentences (r=0.17) and a minimal correlation for unacceptable sentences (r=0.13). We note two possible reasons for these differences. First, the Likert-scale ratings in Sprouse et al. (2013) are ratings of individual sentences where participants do not see both conditions for an item. As such, while we generally capture the acceptability differences found in the standard Likert scale task, it is possible that our GRIS task does not reflect the same cognitive processes that participants used for the Likert-involved tasks in Sprouse et al. (2013). Second, while both our ratings & the Sprouse et al. ratings are z-scored by-participant, we used a 5-point Likert scale and they used a 7-point Likert scale. While z-scoring removes some of the kinds of scale biases found when comparing different scale sizes, the underlying distributions of the ratings may be different (as participants may use scale extremities according to different scales). In our study, we find that most participants rate towards the extremes, with participants preferring the higher end of the scale (participants include a rating of either 4 or 5 in 100% of trials, while they include either a 1 or 2 rating in only 92% of trials). Unfortunately, we are unable to access the raw Likert ratings of Sprouse et al. (2013) to compare the raw rating distributions.

However, Sprouse et al. (2013) also conduct a series of forced-choice (FC) tasks with their stimuli: participants saw both items within each condition and chose which sentence they found to be more acceptable, a more similar task to our results than the one-sentence Likert-rating task. As such, we can analyze the FC data to see whether their data are similarly skewed as our data.

Results are visualized in Figure D.1. We find that our data strongly correlate with the results of the FC data, where the distribution of GRIS ratings for both acceptable and unacceptable sentences align with the general distribution of ratings in the Sprouse et al. (2013) FC selection ratings. In both experiments, participants display strong judgments.

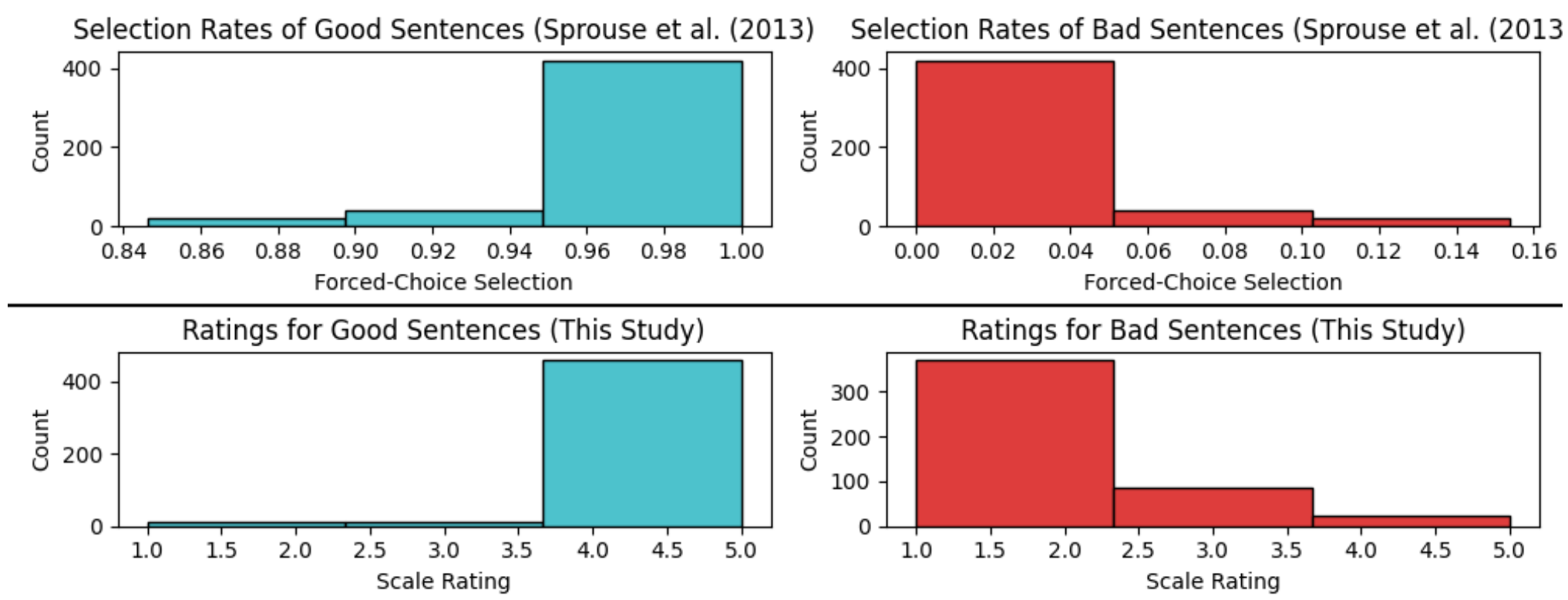

Figure D.1. Comparisons between forced-choice selection rates from Sprouse et al. (2013) and GRIS ratings from our study. Rows indicate experimental study; columns indicate acceptability group.
